# Different Temperature and UV Patterns Modulate Berry Maturation and Volatile Compounds Accumulation in *Vitis* sp.

**DOI:** 10.3389/fpls.2022.862259

**Published:** 2022-06-30

**Authors:** Francisco Campos-Arguedas, Guillaume Sarrailhé, Paméla Nicolle, Martine Dorais, Nicholas J. B. Brereton, Frederic E. Pitre, Karine Pedneault

**Affiliations:** ^1^Department of Science, Université Sainte-Anne, Church Point, NS, Canada; ^2^Centre de Recherche et d'Innovation sur les Végétaux, Département de Phytologie, Université Laval, Québec, QC, Canada; ^3^Institut de Recherche en Biologie Végétale, Université de Montréal et Jardin botanique de Montréal, Montréal, QC, Canada

**Keywords:** fruit aroma, secondary metabolites, gas chromatography-mass spectrometry, climate change, berry ripening, abiotic stress

## Abstract

Volatile compounds (VCs) in grapevine berries play an important role in wine quality; however, such compounds and vine development can be sensitive to environmental conditions. Due to this sensitivity, changes in temperature patterns due to global warming are likely to further impact grape production and berry composition. The aim of this study was to determine the possible effects of different growing-degree day accumulation patterns on berry ripening and composition at harvest. An experimental field was conducted using *Vitis* sp. L'Acadie blanc, in Nova Scotia, Canada. Using on-the-row mini-greenhouses, moderate temperature increase and reduced ultraviolet (UV) exposure were triggered in grapevines during pre-veraison (inflorescence to the beginning of berry softening), post-veraison (berry softening to full maturity), and whole season (inflorescence to full maturity), while controls were left without treatment. Free and bound VCs were extracted from berries sampled at three different phenological stages between veraison and maturity before analysis by gas chromatography–mass spectrometry (GC-MS). Berries from grapevines exposed to higher temperatures during early berry development (pre-veraison and whole) accumulated significantly higher concentrations of benzene derivatives 2-phenylethanol and benzyl alcohol at harvest, but lower concentrations of hydroxy-methoxy-substituted volatile phenols, terpenes, and C_13_-norisoprenoids than the control berries. These results illustrate the importance of different environmental interactions in berry composition and suggest that temperature could potentially modulate phenylpropanoid and mevalonate metabolism in developing berries. This study provides insights into the relationships between abiotic conditions and secondary metabolism in grapevine and highlights the significance of early developmental stages on berry quality at harvest.

## Introduction

Understanding the impact of environmental change (e.g., temperature, altered solar radiation, and water availability) on plants and developing adaptation strategies to climate change are essential to maintaining the productivity of cultivated lands in forestry and agriculture. According to projections from the Intergovernmental Panel on Climate Change (IPCC), global increases in temperature will reach 1.4–4.4°C by 2100 depending on greenhouse gas emissions (Masson-Delmotte et al., 2021). In Eastern Canada, mean temperatures are predicted to increase from 2 to 4°C in the summer and 1.5 to 6°C during the winter (Vasseur and Catto, 2008), leading to a northward expansion of growing degree day (GDD) ≥ 1,200 by 400–600 km by 2099 (King et al., 2018).

Temperature is considered one of the main factors influencing the phenology of grapevine (*Vitis* sp.) (Keller, 2015), and variations in temperature during the developmental and ripening phases have an impact on the biosynthesis of primary and secondary metabolites, specifically during berry growth (Mira de Orduña, 2010; González-Barreiro et al., 2015). Temperature rises due to global warming have been reshaping the dynamics of grapevine harvest and wine production worldwide by globally increasing seasonal heat accumulation (Santos et al., 2020; Venios et al., 2020), a variable often assessed through the sum of GDDs. Increases in GDD accumulation have been shown to accelerate plant development and advance grapevine phenology (leading to earlier harvest dates), shorten the time between bud break and flowering, increase the concentration of sugars, and decrease the acidity content of berries (Mira de Orduña, 2010; van Leeuwen and Darriet, 2016; Cameron et al., 2021). However, in addition to temperature rises, larger temperature ranges throughout the growing season could also have a potential impact on plant growth, phenology, and metabolism (Barnuud et al., 2014). Less is known about the impact of these new seasonal temperature patterns on grapevine development and fruit biochemistry.

Grapevine berry production follows a double sigmoidal growth pattern involving a developmental phase (I; berry formation) that goes from flowering to fruit softening, and a ripening phase (II) that goes from berry softening to fully mature fruit, separated by a short lag phase (Keller, 2015). The first phase (hard, green berries) is characterized by cell division and elongation along with the accumulation of proanthocyanidins (tannins) and organic acids (malic and tartaric acids). During the second phase, called veraison, berries soften and organic acids break down, while sugars and many secondary metabolites, such as terpenes, C_13_-norisoprenoids, and other volatile compounds (VCs), accumulate (Jackson, 2008; Keller, 2015). Variation in abiotic conditions, including temperature, can influence the biosynthesis of these metabolites (Mira de Orduña, 2010; González-Barreiro et al., 2015).

Volatile compounds include different chemical families, such as aliphatic alcohols, aliphatic aldehydes, monoterpenes, benzene derivatives, and C_13_-norisoprenoids, among others (Lund and Bohlmann, 2006; Dunlevy et al., 2009; Roubelakis-Angelakis, 2009). In the fruit, most VCs are bound to a sugar moiety (glycosylated) and a fraction is present as free (aglycon). VCs are involved in plant development and both biotic and abiotic stress defense signaling (Dudareva et al., 2013; Vivaldo et al., 2017). As such, the biosynthesis of VCs can be influenced by environmental changes such as seasonal conditions and agricultural practices, and these factors can, therefore, influence the grape and wine aroma profile of the same cultivars within the same area (Lund and Bohlmann, 2006; Dunlevy et al., 2009; Moreno and Peinado, 2012).

The biosynthesis of VCs as a response of plants exposed to high temperatures varies with the intensity and time of exposure. Previous studies on the effect of temperature on VC accumulation have found that compound production and emission increase at high temperatures (Guenther et al., 1993; Copolovici and Niinemets, 2016; Rienth et al., 2021); however, in grapevines, this effect is not consistent (Selmar and Kleinwächter, 2013; Rienth et al., 2016; Lecourieux et al., 2017; Pastore et al., 2017) as many other variables, such as radiation, cultivar, and terroir, can play an important role (Mira de Orduña, 2010; Rienth et al., 2021). In other model plants, such as *Camellia sinensis*, compounds, such as 2-phenylethanol (a product of the shikimate pathway), have been favored at high temperatures (Huang et al., 2001; Shu et al., 2021) and in *Polygonum minus*, the biosynthesis of certain terpenes and aldehydes was related to elevated temperatures (Goh et al., 2016).

Variations in temperature due to climate change and the evolution of VCs during ripening can affect berry quality (Bonada et al., 2015). Several studies have assessed the effects of temperature in controlled conditions such as greenhouses or growth chambers (Salazar Parra et al., 2010; Luchaire et al., 2017; Kizildeniz et al., 2018) and approaches that are valuable in providing important information on the fundamental responses to abiotic stressors and grapevine's physiology and development. However, interactions with other factors, such as rainfall, radiation, humidity, and soil management, can be better explored under field conditions.

Several studies have used experimental field trials where vines have been exposed to different environmental conditions, and plant physiology, berry quality, and berry yield responses were assessed (Sadras and Soar, 2009; Soar et al., 2009; Sadras and Moran, 2012; Sadras et al., 2012a,b, 2013; Bonada et al., 2015). A similar field trial was used here to test whether changes in temperature patterns at different stages of berry development (pre-veraison, veraison, and post-veraison) or a global rise in temperature (whole season) could lead to modifications of the profile of VCs in mature berries. On-the-row mini-greenhouses were used at different berry developmental stages of *Vitis* sp. cv L'Acadie blanc to alter GDD accumulation patterns along the growing season. Berries were sampled at three phenological stages around full maturity, and the composition of free VC (FVC) and glycosylated VC (GVC) was analyzed.

## Materials and Methods

### Treatments and Experimental Design

**Figure 1:**
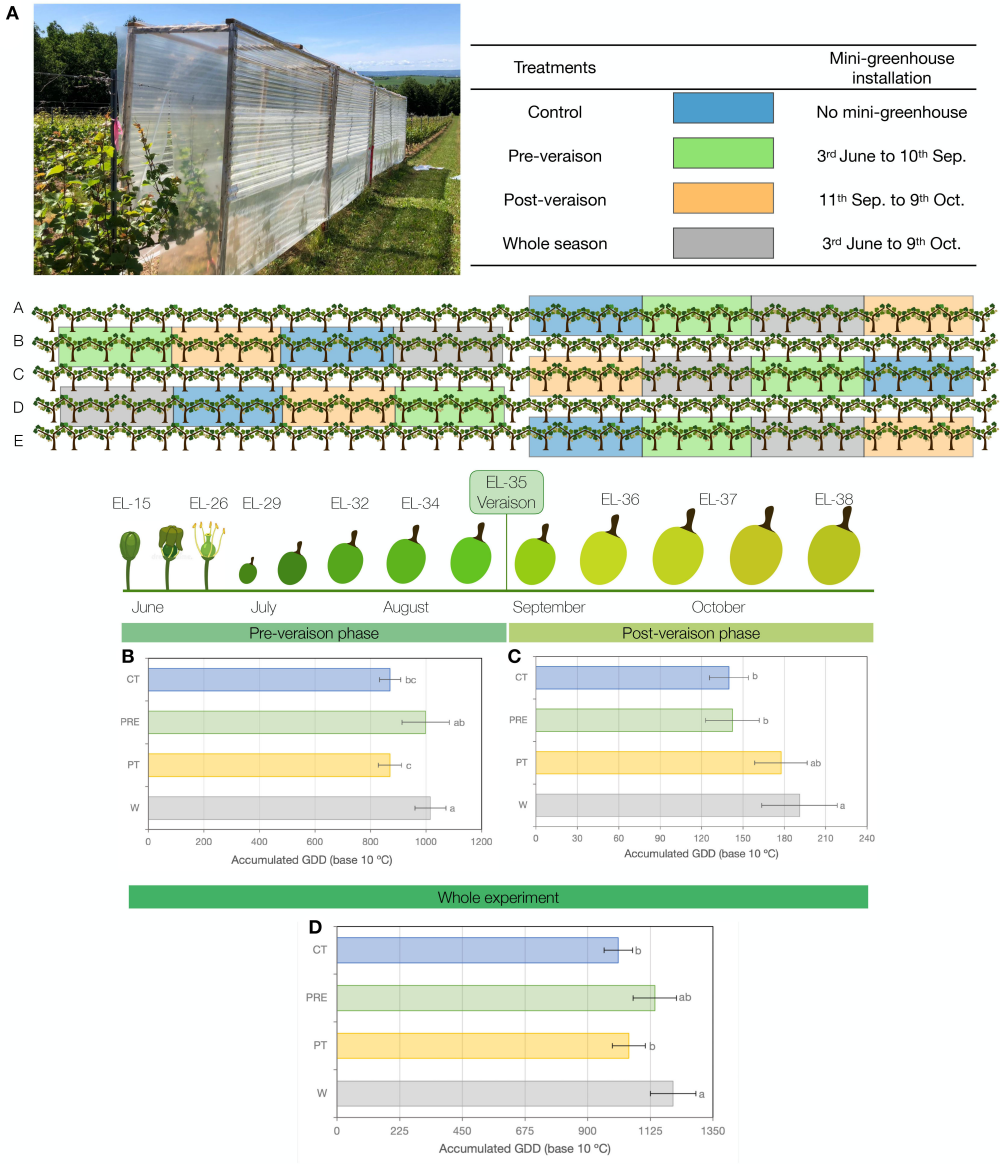
**(A)** Mini-greenhouse, temperature treatments over the growing season, and experimental design. **(B)** Accumulation of growing degree days (GDD; based on 10°C) during the experiment. Pre-veraison phase (from 3 June 2020 to 10 September 2020). **(C)** Post-veraison phase (from 11 September 2020 to 9 October 2020). **(D)** Whole experiment (from 3 June 2020 to 9 October 2020). Values are means ± SD (*n* = 5 for Whole, Pre, and PT; *n* = 3 for CT). For each graph, values with different letters indicate significant differences between treatments (Tukey's test at *p* ≤ 0.05).

The experiment was carried out in a commercial vineyard located in the Gaspereau Valley, Wolfville, NS, Canada (45°4'19, 56”N, 64°17'44.7108”W) during the 2020 growing season. The 11-year-old, self-rooted “L'Acadie blanc” (Cascade X Seyve-Villard 14-287) was used. The vines were pruned to 12 buds per vine and trained in vertical shoot position. Row spacing and vine spacing were 6.0 and 1.2 m, respectively. A complete randomized block design, including five blocks and four treatments, was used. Each block consisted of four plots with four consecutive vines each (Figure 1A). To avoid edge effects, border rows were included between blocks (Figure 1).

Treatments consisted of temperature increases triggered by polycarbonate mini-greenhouses (Figure 1A) installed on-the-row at specific times and locations. Each structure (4.9 m length × 0.6 m width × 2.4 m height) was covered with polycarbonate panels (Tuftex, Onduline, VA, USA), transmitting 89% of the incident light within the visible spectrum and blocking ultraviolet (UV) radiation below the 380 nm. Due to this UV blocking effect, significant differences in VC accumulation should be considered a result of mini-greenhouse treatment, which includes both altered GDD and UV exposure. Two sides of the structure include covering by a clear polyethylene film cover (A&A Grower Supply) at the bottom, with a 20-cm gap left at ground level, and leaving two sides of the mini-greenhouse open to allow the circulation of air (Figure 1). Holes (~5 mm diameter) were made at the top of the structure to avoid rain accumulation. The modified Eichhorn-Lorenz (EL) system was used to describe phenological stages as described by Coombe (1995). The vines were exposed to four different treatments (Figure 1A), namely, 1. whole season (W): mini-greenhouse installation from the shoot and inflorescence developmental stage at EL-15 (3 June) until harvest at EL-38 (9 October), 2. pre-veraison (Pre): mini-greenhouse installation from EL-15 (3 June) until veraison at EL stage 35 (11 September), 3. post-veraison (PT): mini-greenhouse installation from EL-35 (11 September) until harvest at EL-38 (9 October), and 4. control (CT): without mini-greenhouse. To achieve a target of two clusters per shoot, cluster and shoot thinning were performed prior to veraison to level the number of clusters and the number of shoots in each plant (clusters per plant = 1.70 × shoot number + 1.70, with *r*^2^ = 0.89).

### Climatic Measurements

Temperature and humidity were monitored in each plot. Relative humidity at canopy level was measured hourly by TemLog 20H data logger from Elitech Technology Inc. (Milpitas, CA, USA). Temperature and light (lux) were measured hourly by Hobo Pendant Temperature/Light Data Logger (UA-002-64) from ONSET (Bourne, MA, USA) above the canopy. The following equation is used to calculate the accumulation of degree days (GDD):


$$\begin{array}{l} GDD=\overset{n}{\underset{i=1}{\sum }}T_{i}-T_{b} \end{array}$$

where a base temperature (T_*b*_) of 10°C is used, n is the total number of days in each period, and T_*i*_ is the mean daily temperature (max+min/2).

### Sampling

Berries were sampled at the following stages: EL-36 (“*berries with intermediate brix values*”), EL-37 (“*berries not quite ripe*”), and EL-38 (“*harvest-ripe*”) (Coombe, 1995). The samples were immediately frozen with liquid nitrogen, transported in dry ice, and stored at −80°C until analyzed. Each sample consisted of 6–8 clusters randomly picked on all four vines of each treatment.

### Physicochemical Analysis of Grapes

For each experimental unit, 200 fresh berries were weighted, hand-crushed, and filtered through a sieve. The concentration of total soluble solids (TSS; °Brix) of the must was measured with a PAL-1 pocket refractometer (Atago Co., Ltd., Tokyo, Japan). Titratable acidity (TA; as g L^−1^ of tartaric acid equivalent) was quantified with an HI 84502 titratable total acidity mini-titrator (Hanna Instruments. Woonsocket, RI, USA), as follows: 2 ml of juice were added and diluted to a final volume of 50 ml (specifications for high range TA; 4.0–25.0 g L^−1^ of tartaric acid) and titrated with the HI 84502-50 titrant solution (NaOH) to a final pH of 8.2. pH was measured using an MP 200 pH meter (Mettler Toledo, Columbus, OH, USA).

### Analysis of Volatile Aroma Compounds

#### Chemicals

Ethanol (97%), methanol (HPLC grade), dichloromethane (HPLC grade) n-Hexane (99%), and insoluble polyvinylpolypyrrolidone (PVPP) were purchased from Millipore Corporation (Billerica, MA, USA). n-Pentane (HPLC grade) and citric acid (anhydrous) were purchased from Anachemia (Mississauga, ON, CA). Sodium sulfate anhydrous was purchased from VWR (Solon, OH, CA) and sodium phosphate dibasic was purchased from Spectrum Chemicals (Gardena, CA, USA). Rapdidase Revelation Aroma enzyme (a mixture of pectinases and glycosidases) was purchased from ScottLabs Canada (Niagara-on-the-lake, ON, CA). Internal standards (±)-2-octanol (≥ 99.5%) and nonyl-ß-∂-glucopyranoside (≥ 97%) were purchased from Sigma Aldrich (Oakville, ON, CA). All the other authentic standards used to determine the retention time are listed in Supplementary Table 1.

#### Sample Extraction

Free and glycosylated volatile compounds were extracted from 200 g of grape berries. The berries were thawed at 4°C overnight. The juice was extracted, filtered through cheesecloth, and centrifuged at 4°C at 4,234 g for 20 min. The juice was again filtered on cotton and 1 g of PVPP was added per 100 g of juice and stirred for 20 min. The juice was vacuum filtered with Whatman paper (grades 3, 4, and 5). Fractions of 100 ml of juice were taken and 100 ml of distilled water was added, as well as 100 μl of 2-octanol (230 mg L^−1^ in ethanol) and 0.45 ml of nonyl-β-∂-glucopyranoside (1,000 mg L^−1^ in ethanol/water, 50:50) were used as internal standards (Paolini et al., 2018).

Volatile compounds were extracted by adsorption on Isolute ENV + solid-phase extraction (SPE) cartridges (500 mg, 6 ml) purchased from Biotage (Charlotte, NS, USA). The cartridges were conditioned with 20 ml of methanol and 20 ml of distilled water at a flow rate of 1 ml min^−1^. The sample was loaded onto the cartridge and then washed with 50 ml of distilled water. The FVCs were then eluted with 25 ml of dichloromethane (Schneider et al., 2004). The extract was filtered through anhydrous sodium sulfate and 50 ml of pentane was added (to a ratio of dichloromethane: pentane, 1:2), and the solution was concentrated to 200 μl using a Kuderna-Danish at 35–40°C (Schneider et al., 2004). The extract was stored at −80°C until gas chromatography–mass spectrometry (GC-MS) analysis.

The GVC fraction was eluted with 25 ml of methanol and evaporated to dryness under nitrogen at 45°C. All traces of FVCs were removed with dichloromethane: pentane (1:2). The remaining residue was then dissolved in 500 μl of phosphate/citrate buffer at pH 5 (Lanaridis et al., 2016). A volume of 200 μl of enzyme solution from Rapdidase Revelation Aroma (70 mg ml^−1^ in phosphate/citrate buffer, pH 5) was added (Schneider et al., 2004; Crespo et al., 2017), and the solution was kept at 40°C for 24 h. Once the enzymatic reaction was completed, internal standard 2-octanol (25 μl; 230 mg L^−1^ in ethanol) was added and the volatile fraction was extracted with dichloromethane: pentane (1:2) solution; the sample was concentrated at 200 μl using a Kuderna-Danish at 35°C−40°C. The extract was stored at −80°C until GC-MS analysis.

#### GC-MS Analysis

Compounds were separated on an HP-5MS Ultra Inert chromatographic column (30 m × 250 μm × 0.25 μm). Injection volumes were 1 μl and it was carried out in splitless mode. Helium was used as the carrier gas. The oven temperature was 40°C for 2 min, increased to 240°C at a rate of 3.5°C min^−1^, maintained at 240°C for 2 min, increased to 250°C at a rate of 20°C min^−1^, and held isothermal for 5 min at 250°C. Compounds were analyzed using an Agilent 7890A gas chromatography instrument coupled to an Agilent 5975 mass selective detector and a flame ionization detector using a splitter (Agilent Technologies, Santa Clara, CA, USA) and equipped with the ChemStation software for data acquisition and analysis. Compound identification was performed by mass spectra comparison, by NIST MS Search 2.0 library, and by retention indexes (Slegers et al., 2015), and for certain compounds, identification was possible using authentic standards (Supplementary Table 1). As commonly used in the analysis of aroma compounds (Azzolini et al., 2012; Ghaste et al., 2015), the response of the internal standard 2-octanol was used to normalize the peak area and to make a relative estimation of the concentration of the identified compounds, considering a response factor equal to 1.00.

### Statistical Analysis

All statistical analyses were performed using the R software (RCoreTeam, 2022). Analysis of variance (ANOVA) was carried out for the climatic measurements, VCs, TSS, pH, berry weight (g), and TA. Means were compared using Tukey's *post hoc* honestly significant difference (HSD) at *p* ≤ 0.05. Repeated measure ANOVA was also performed, and means were also compared using the Tukey HSD test to determine the significance of the VCs in response to the study factors (temperature and phenological stage). Principal component analysis (PCA) was carried out for compounds that were significant at *p* ≤ 0.05, *p* ≤ 0.01, and *p* ≤ 0.001 in terms of treatment and phenological stage. Pearson's correlations between environmental variables (GDD) and berry maturity variables (TSS, TA, and pH) were estimated and considered significant at *p* ≤ 0.001.

## Results

### Evolution of the Growing Degree-Days Pattern and the Climatic Conditions

The mini-greenhouses used in this experiment efficiently modulated mean temperatures (Supplementary Figure 1) and GDD accumulation during the pre-veraison phase (Pre treatment), post-veraison phase (PT treatment), whole experiment (W treatment), and/or CT (treatment without mini-greenhouses) (Figure 1). GDD accumulation was higher in the Pre and W treatments during the pre-veraison phase from 3 June 2020 to 10 September 2020, accumulating 999 and 1,016 GDD, respectively, compared to the 870 GDD and 871 GDD accumulated in PT and CT treatments, respectively (Figure 1B). During the post-veraison phase from 11 September 2020 to 9 October 2020, the W treatment accumulated more GDD than the Pre and CT treatments (Figure 1C). During the whole experiment (3 June 2020 to 9 October 2020), the GDD was significantly higher in the W treatment with 1,207 GDD compared to 1,048 GDD and 1,010 GDD in the PT and CT treatments, respectively, while no significant difference was observed between the control and Pre or PT treatments (Figure 1D). Canopy temperature for W and PRE shows higher mean values at the beginning of the season, in line with the establishment of the mini-greenhouses (Supplementary Figure 1). Lately, during the season, specifically in September, the patterns of mean temperature switched from Pre to PT, being the W and PT the treatments showing higher mean temperatures during the last 2 months of the experiment (refer to Supplementary Figure 1).

### Berry Growth and Juice Basic Metrics

Berries were sampled at three different phenological stages around full ripeness (EL-36, EL-37, and EL-38). Juice metrics and berry weight showed significant differences between phenological stages (Table 1). The highest value of TSS was found at full ripeness, 22.4 °Brix at EL 38, when compared to EL-37 (21.4 °Brix) and EL-36 (19.5 °Brix), which were also significantly different from each other. TA (g·L^−1^ tartaric acid eq) showed a similar trend in all treatments, as the values decreased with berry maturity, with the lowest value of 8.55 g L^−1^ tartaric acid eq reached at EL 38. Berry weight increased during ripening until EL-38 was reached, with a mean value of 1.2 g berry^−1^.

**Table 1:** Effects of the four mini-greenhouse treatments (CT, control; Pre, pre-veraison; PT, post-veraison; W, whole season) on berry weight (g), total soluble solids (°Brix), pH, and titratable acidity (g L^−1^ of tartaric acid eq) of L'Acadie blanc berries, vintage 2020.

**Variables^**a**^**	**CT**	**Pre**	**PT**	**W**	***p*-value**
Berry weight	1.14 ± 0.08	1.13 ± 0.11	1.16 ± 0.07	1.13 ± 0.09	0.774
Total soluble solids	20.39 ± 1.64a	21.42 ± 1.23ab	20.89 ± 1.43ab	21.79 ± 1.47b	0.0543
pH	3.01 ± 0.09	2.96 ± 0.08	3.02 ± 0.10	2.95 ± 0.14	0.179
Titratable acidity	9.89 ± 1.50	10.14 ± 1.27	9.69 ± 1.16	10.22 ± 1.46	0.696

a *Data are means ± standard deviation of n = 15. For each variable, values with different letters indicate significant differences between treatments according to Tukey's test at p < 0.1*.

The mini-greenhouse treatments had less impact on berry weight and juice metrics (Table 2). Productivity varied from 1.86 to 2.44 kg vine^−1^ between different treatments but was not significantly different (Supplementary Table 2).

**Table 2:** Effects of the phenological stage (EL-36, EL-37, and EL-38) on berry weight (g), total soluble solids (°Brix), pH, and titratable acidity (g L^−1^ of tartaric acid eq) of *Vitis* sp. cv. L'Acadie blanc berries, vintage 2020.

**Variables^**a**^**	**EL-36**	**EL-37**	**EL-38**	***p*-value**
Berry weight	1.10 ± 0.09a	1.15 ± 0.06ab	1.17 ± 0.09b	0.0209
Total soluble solids	19.50 ± 1.17a	21.45 ± 0.72b	22.43 ± 0.71c	<0.001
pH	2.88 ± 0.10a	3.02 ± 0.06b	3.05 ± 0.05b	<0.001
Titratable acidity	11.37 ± 0.77c	10.05 ± 0.70b	8.55 ± 0.53a	<0.001

a *Data are means ± standard deviation of n = 20. For each variable values with different letters indicate significant differences between treatments according to Tukey's test at p < 0.05*.

Berries from the W treatment had a value of 21.8 °Brix, a higher TSS compared to the control (CT, 20.4 °Brix; Table 2) although pH, TA, and berry weight were similar. CT and Post treatments had visibly increased red coloration compared to Whole and Pre treatments (Supplementary Figure 3).

Berry pH increased as berries matured and was negatively correlated with TA (Supplementary Figure 2). Similarly, TA was also negatively correlated with TSS (Supplementary Figure 2), and TSS was positively correlated with pH (Supplementary Figure 2). Only TSS was significantly correlated with GDD (r^2^ = 0.49, *p* < 0.001; Supplementary Table 3).

### Influence of Ripening and Mini-Greenhouse Treatments on Volatile Compounds

A total of 46 and 53 compounds were detected in the FVC and GVC fractions, respectively, and were classified into nine classes (Tables 3, 4), namely, (1) aliphatic alcohols, (2) aliphatic aldehydes, (3) aliphatic acids, (4) terpenes, (5) C_13_-norisoprenoids, (6) aliphatic esters, (7) volatile phenols, (8) benzene derivatives, and (9) other volatiles. Among these, aliphatic esters were only detected as FVC and terpenes, and C_13_-norisoprenoids were only detected as GVC. Aliphatic aldehydes represented the largest proportion of FVC, with a mean concentration of 11.74 μg g^−1^ FW. The GVC fraction was mainly composed of benzene derivatives (2,254 ng g^−1^ FW, on average) followed by C_13_-norisoprenoids (973 ng g^−1^ FW, on average) and terpenes (895 ng g^−1^ FW, on average).

**Table 3:** Impact of the temperature treatments (Control; Pre, pre-veraison; Post, post-veraison; Whole, whole season) on the profile of free volatile compounds (ng·g^−1^ FW) of berries from *Vitis* sp. cv. L'Acadie blanc in 2020.

**Compounds^**a**^**	**CT**	**Pre**	**PT**	**W**	***p*-value**
**Aliphatic alcohols**					
*(Z)*-2-Penten-1-ol	41.7 ± 10.8	34.2 ± 13.1	34.5 ± 13	30.9 ± 9.2	0.0941
**1-Hexanol**	170 ± 240	139 ± 139	142 ± 165	107 ± 113	0.7940
2-Hexanol	3.0 ± 1.7	2.1 ± 0.9	3.3 ± 2.0	3.2 ± 2.6	0.6600
*(E)*-2-hexen-1-ol	408 ± 110b	336 ± 172ab	376 ± 81.4ab	272 ± 134 a	0.0330
2,4-Dimethyl-1-heptanol	382 ± 235b	220 ± 160ab	290 ± 233ab	174 ± 139 a	0.0443
2,6-Dimethyl-2-octanol	11.7 ± 13.0	12.8 ± 7.2	15.8 ± 10.8	14.0 ± 9.6	0.8020
3,7,11-Trimethyl-1-dodecanol	27.3 ± 39.2	10.7 ± 9.0	16.0 ± 13.9	14.9 ± 14.7	0.3120
*Sum*	988 ± 234c	732 ± 244ab	837 ± 194bc	595 ± 200 a	<0.0001
**Alpihatic aldehydes**					
Hexanal	2 608 ± 325	2 289 ± 367	2 445 ± 591	2 392 ± 234	0.1890
2-Hexenal	86.1 ± 21.5	83.2 ± 13	80.4 ± 16	80 ± 23	0.7950
*(E)*-2-Hexenal	9,876 ± 1 019	8,918 ± 986	8,924 ± 1,524	9 082 ± 784	0.0660
2,3,4-Trimethyl-hex-3-enal	2.9 ± 0.7	41.9 ± 67.1	2.9 ± nd	3.8 ± nd	0.7500
*(E,E)*-2,4-Hexadienal	3.2 ± 0.8	2.7 ± 1.2	2.8 ± 1.2	3.1 ± 0.7	0.6510
Nonanal	9.4 ± 7.8	10.7 ± 9.2	8.6 ± 3.8	10.9 ± 6.8	0.8060
*(E,E)*-2,6-Nonadienal	10.9 ± 2.9	13.2 ± 4.9	12.5 ± 4.2	10.4 ± 3.8	0.1960
*(E)*-4-Undecenal	1.4 ± 0.4	2.0 ± 1.5	1.6 ± 0.8	1.6 ± 0.7	0.4690
*Sum*	12 595 ± 1,308	11 325 ± 1,333	11,474 ± 2,062	11,579 ± 946	0.0856
**Aliphatic acids**					
2-Propenoic acid pentyl ester	98.6 ± 29b	66.2 ± 35.9a	80.6 ± 32.8ab	70.9 ± 32.8ab	0.0446
**Butyric acid-5-hexenyl ester**	11.0 ± 5.0b	7.4 ± 2.1a	8.1 ± 2.5ab	8.5 ± 3.0ab	0.0398
Butyric acid octyl ester	12.6 ± 5.6	14.9 ± 7.4	14.8 ± 7.7	16.7 ± 9.6	0.5450
Hexanoic acid	32.0 ± 18.9	27.6 ± 10.9	29.4 ± 17.3	26.1 ± 8.2	0.7040
2-Ethyl-hexanoic acid	3.1 ± 2.2	2.5 ± 1.2	3.0 ± 1.3	2.5 ± 1.1	0.8240
*(E)*-2-Hexenoic acid	34.7 ± 16.9	31.1 ± 19.5	43.7 ± 22.7	24.8 ± 19.5	0.3680
Heptanoic acid	4.4 ± 2.1	3.6 ± 0.9	4.2 ± 2.8	4.5 ± 1.8	0.6540
Octanoic acid	12.2 ± 3.7	11.4 ± 6.2	12.8 ± 6.3	13.0 ± 6.1	0.8790
7-Oxooctanoic acid	10.5 ± 6.6	11.1 ± 4.3	10.7 ± 5.5	11.2 ± 3.2	0.9810
*Sum*	197 ± 52.5	156 ± 53.5	179 ± 57.7	164 ± 56.3	0.1910
**Aliphatic esters**					
2-Butoxy-ethanol	4.2 ± 1.4	4.2 ± 1.3	4.4 ± 1.5	4.7 ± 1.1	0.6940
2,2-Butoxyethoxy-ethanol	14.4 ± 7.2	16.8 ± 8.6	14.4 ± 6.3	19.9 ± 8.6	0.1750
*Sum*	18.3 ± 7.8	20.5 ± 9.1	18.8 ± 7.5	24.7 ± 9.2	0.1690
**Volatile phenols**					
**Methyl salicylate**	7.3 ± 4.1a	14.9 ± 7.8b	10.1 ± 6.4ab	8.9 ± 5.8ab	0.0105
Vanillin	27.5 ± 16.1	32.9 ± 28.8	23.9 ± 13.1	30 ± 12.1	0.6030
4-Hydroxy-3,5-dimethoxy-benzaldehyde	17.0 ± 12.7	15.2 ± 15.1	14.7 ± 9.7	12.1 ± 7.5	0.7180
*Sum*	51.8 ± 24.5	63.0 ± 43.9	48.7 ± 16.2	51.1 ± 15.0	0.4970
**Benzene derivatives**					
Benzyl alcohol	35.0 ± 9.4	33.1 ± 13.5	27.6 ± 14.7	29.4 ± 12.8	0.3800
Phenylethanal	24.6 ± 21.8	12.1 ± 7.2	26.4 ± 24.8	15.3 ± 11.8	0.0908
*p*-Tolualdehyde	10.8 ± 13.3	9.6 ± 11	10.3 ± 11.3	13.7 ± 14.7	0.8610
Benzophenone	11.4 ± 1	11.8 ± 1	11.8 ± 2.1	12.0 ± 1.5	0.7190
2-Phenoxy-ethanol	4.7 ± 0.8	6.3 ± 3.8	5.6 ± 2.2	5.6 ± 2.4	0.4470
*Sum*	85.1 ± 23.2	67.9 ± 23.2	79.2 ± 37.6	73.8 ± 27.0	0.3980
**Other volatiles**					
3,4,4-Trimethyl-2-hexene	7.0 ± 0.2	6.3 ± 0.1	6.9 ± 1.2	6.5 ± 0.2	0.6760
**Heptane**	50.8 ± 21.9	48 ± 20.6	49.2 ± 24.4	57.1 ± 17.4	0.6500
4-Methyl-heptane	12.6 ± 9.7	8.1 ± 2.8	16.4 ± 14.7	11.8 ± 10.2	0.3840
2,4-Dimethyl-heptane	6.3 ± 6.4	3.8 ± 2.0	6.8 ± 4.8	4.8 ± 3.5	0.4750
2,4-Dimethyl-1-heptene	55.4 ± 28.4	34.8 ± 19.7	44.4 ± 14.1	23.3 ± 15.3	0.0582
4-Methyl-nonane	5.5 ± 3.0	4.8 ± 1.6	5.0 ± 2.6	5.4 ± 1.4	0.8140
4-Propyl-3-heptene	2.4 ± 0.2	2.1 ± 0.7	2.7 ± 1.0	2.4 ± 0.3	0.6050
2,2-Dimethyl-3-octene	5.9 ± 2.6	4.8 ± 2.0	5.8 ± 2.9	5.4 ± 2.0	0.6090
2,3,3-Trimethyl-1,7-octadiene	4.2 ± 3.8	2.4 ± 1.1	4.1 ± 4.5	4.4 ± 3.0	0.8940
Decane	28.6 ± 13.3b	17.5 ± 10.1ab	25.4 ± 11.8ab	16.7 ± 9.7a	0.0433
4-Ethyl-decane	3.1 ± 1.2	3.7 ± 1.5	5.3 ± 3.5	3.3 ± 2.7	0.2660
*y*-Undecalactone	3.6 ± 3.1	4.5 ± 5.6	3.7 ± 2.6	3.3 ± 1.6	0.8370
**Total**	14,055 ± 1,362b	12,455 ± 1,359a	12,750 ± 1 971ab	12,607 ± 954a	0.0147

*Variables showing a significant interaction between treatments and phenological stages are in bold (refer to Supplementary Table 4 for repeated measure ANOVA and Tuckey's comparison test).*

a *All compounds were quantified as 2-octanol equivalents. Values are means ± standard deviation of 15 biological replicates. For each treatment, values with different letters indicate significant differences according to Tukey's test at p < 0.05. ns, not significant; nd, not determined. Repeated measure ANOVA was also carried out to detect possible interactions between temperature; treatments and phenological stages (Supplementary Table 6)*.

**Table 4:** Impact of the temperature treatments (Control; Pre, pre-veraison; Post, post-veraison; Whole, whole season) on the profile of glycosylated volatile compounds (ng · g^−1^ FW) of berries from *Vitis* sp. cv. L'Acadie blanc in 2020.

**Compounds^**a**^**	**CT**	**Pre**	**PT**	**W**	***p*-value**
**Aliphatic alcohols**					
3-Methyl-1-butanol	26.8 ± 5.6	25.5 ± 7.3	22.7 ± 9.6	24.4 ± 5.5	0.4786
2-Methyl-1-butanol	24.4 ± 8.5	24.2 ± 10.3	19.0 ± 8.5	19.5 ± 7.6	0.1838
2-Methyl-2-buten-1-ol	13.4 ± 5.2	13.4 ± 4.0	13.1 ± 5.5	12.3 ± 4.2	0.9034
3-Methyl-3-buten-1-ol	48.5 ± 10.2	46.5 ± 10.1	41.0 ± 11.4	46.1 ± 10.0	0.2540
1-Pentanol	9.0 ± 3.8	7.8 ± 2.3	7.8 ± 4.2	6.4 ± 2.2	0.1790
**1-Hexanol**	25.3 ± 15.7	20.3 ± 8.9	28.9 ± 23.9	17.7 ± 7.4	0.1991
3-Hexen-1-ol	15.2 ± 5.3c	8.3 ± 3.4a	13.8 ± 5.3bc	9.8 ± 5.4 ab	0.0008
*Sum*	163 ± 41.9	146 ± 35.0	146 ± 60.5	136 ± 30.8	0.4250
**Aliphatic aldehydes**					
Hexanal	11.8 ± 6.3	11.1 ± 4.6	12.7 ± 6.3	8.6 ± 2.4	0.1738
**(*****E*****)-2-Hexenal**	32.1 ± 16.9ab	23.1 ± 9.8ab	36.6 ± 27.8b	19.5 ± 11.1a	0.0422
*Sum*	43.9 ± 21.1ab	34.2 ± 12.3ab	49.3 ± 32.1b	28.1 ± 11.6a	0.0349
**Aliphatic acids**					
(*Z*)-9-Octadecenoic acid	28.8 ± 13.2	28.3 ± 10.5	32.8 ± 18.2	31.7 ± 13.5	0.7834
Tetradecanoic acid	49.5 ± 20.1	41.4 ± 20.1	45.0 ± 23.3	43.8 ± 15.0	0.7253
*Sum*	78.3 ± 27.7	69.6 ± 23.3	77.7 ± 36.5	75.5 ± 18.6	0.8153
**Mono- and sesquiterpenes**
**(*****Z*****)-Linalool oxide**	63.8 ± 26.3b	38.3 ± 11.7a	45.6 ± 14.5a	36.1 ± 12.8a	0.0002
(*E*)-Linalool oxide	37.1 ± 9.7b	29 ± 4.1a	33.2 ± 5.3ab	30.6 ± 5.5a	0.0073
**Linalool oxide pyranoid**	56.5 ± 22.4b	32.9 ± 11.7a	43.6 ± 12.1 ab	32.2 ± 12.3a	0.0001
Linalool	11.2 ± 13.5	5.7 ± 4.7	9.0 ± 11.1	5.3 ± 4.8	0.2699
**Hotrienol**	110 ± 81.8b	53.4 ± 27.4a	58.3 ± 31.9 a	45.9 ± 35.7a	0.0027
Nerol	20.2 ± 12.1b	10.0 ± 7ab	20.4 ± 19.4b	9.2 ± 8.0a	0.0190
**Lavandulol**	17.1 ± 3.9	15.6 ± 2.9	18.7 ± 8.0	16.7 ± 1.6	0.3489
(*E*)-8-Hydroxylinalool	101 ± 34.8b	58.9 ± 21.4a	77 ± 22a	59.9 ± 17.7a	<0.0001
**(*****Z*****)-8-Hydroxylinalool**	839 ± 472b	414 ± 225a	632 ± 235 ab	439 ± 202a	0.0009
Linalyl isobutyrate	32.3 ± 15.4	24.8 ± 7.7	26.0 ± 6.9	26.0 ± 8.5	0.1844
2,6-Dimethyl-2,6-Octadiene-1,8-diol	8.8 ± 10.1	7.0 ± 6.2	8.8 ± 8.5	6.4 ± 5.5	0.7737
Nerolidol	14.4 ± 4.5b	9.4 ± 2.5a	11.9 ± 3.5 ab	8.9 ± 2.7a	<0.0001
**Lilac alcohol C**	10.7 ± 6.9	6.6 ± 3.7	9.5 ± 4.6	6.8 ± 4.4	0.0817
***Sum***	1,322 ± 656b	705 ± 317a	994 ± 353ab	723 ± 294a	0.0005
**C**_**13**_ **-norisoprenoids**					
3-Hydroxy-β-damascone	172 ± 34.0	148 ± 26.5	152 ± 27.7	142 ± 45.0	0.0940
3-Hydroxy-7,8-dihydro-β-ionol	87.1 ± 37.7	66.7 ± 15	70.2 ± 18.6	72.6 ± 14.7	0.0998
3-Oxo-α-ionol	349 ± 67.3	302 ± 55.2	315 ± 64.3	290 ± 85.4	0.1160
β-Ionol	236 ± 102b	155 ± 37.1a	174 ± 38.9a	164 ± 34.9a	0.0021
3-Hydroxy-5,6-epoxy-β-ionone	24.5 ± 5.4	20.3 ± 6.5	23.8 ± 5.0	20.9 ± 4.2	0.0954
3-Oxo-7,8-dihydro-α-ionol	236 ± 39.8b	191.7 ± 31a	206.1 ± 34.6ab	201.1 ± 22.9a	0.0033
Dihydro-3-oxo-β-ionol	21.5 ± 7.8b	15.4 ± 4.2a	18.4 ± 5.0ab	18.4 ± 4ab	0.0332
*Sum*	1,126 ± 269b	899 ± 160a	959 ± 181ab	909 ± 144a	0.0076
**Volatile phenols**					
*p*-Vinylguaiacol	21.3 ± 6.2	17.1 ± 9.1	19.9 ± 10.7	16.4 ± 6.8	0.3397
Eugenol	36.7 ± 16.3	30.9 ± 10.8	36.8 ± 18.2	44.5 ± 14.6	0.1120
Methoxyeugenol	10.8 ± 2.4	10.9 ± 2.6	10.7 ± 3.6	9.8 ± 2.0	0.6660
2-Hydroxy-benzeneethanol	18.8 ± 16.1	11.5 ± 7.2	14.1 ± 7.7	9.9 ± 4.8	0.0867
Isoeugenol	22.4 ± 13.3b	10.3 ± 3.6a	16.6 ± 4.6ab	10.8 ± 2.4a	<.0001
Isovanillyl alcohol	33.6 ± 17.3b	18.8 ± 6.6a	28.4 ± 11.8ab	19.9 ± 8.6a	0.0024
Acetovanillone	30.8 ± 6.0b	25.3 ± 5.0a	29.2 ± 6.1ab	24.3 ± 4.8a	0.0053
**Methyl vanillate**	129 ± 134b	67.4 ± 68.3ab	68.5 ± 21.2ab	53.8 ± 19.6a	0.0445
Methyl 3-hydroxybenzoate	32.0 ± 10.9b	22.4 ± 8.4a	28.2 ± 7.9ab	20.6 ± 8.8a	0.0034
(*E*)-Coniferyl alcohol	56.4 ± 31.9b	29.7 ± 20.2a	42.6 ± 30.3ab	24.2 ± 13.3a	0.0042
Sinapyl alcohol	34.3 ± 27.1b	19.3 ± 9.9ab	22.6 ± 12.0ab	17.0 ± 9.8a	0.0266
Salicyl alcohol	21.0 ± 8.1	18.8 ± 7.5	16.2 ± 6.1	14.8 ± 4.7	0.0693
5-(3-Hydroxypropyl)-2,3-dimethoxyphenol	13.9 ± 9.7	9.5 ± 4.9	10.9 ± 6.1	7.5 ± 2.4	0.0552
2-Hydroxy-4,5-dimethylacetophenone	40.0 ± 12.5	32.2 ± 10.3	31.5 ± 10.0	31.6 ± 8.8	0.0846
4-tert-Butyl-2-methylphenol	53.5 ± 13.8	46.6 ± 10.1	45.4 ± 12.2	48.9 ± 9.6	0.2434
*Sum*	554 ± 250b	371 ± 129a	422 ± 113ab	354 ± 69.0a	0.0034
**Benzene derivatives**					
Benzyl alcohol	1 094 ± 96.6a	1 390 ± 298b	1 004 ± 157a	1 308 ± 182b	<0.0001
2-Phenylethanol	770 ± 85.1a	1 152 ± 143b	756 ± 132a	1 123 ± 163b	<0.0001
3-Tridecyl ester-m-toluic acid	86.1 ± 17.8	71.9 ± 19.4	78.9 ± 18.7	91.8 ± 67.2	0.4968
4-Benzyloxy-3-methoxybenzyl alcohol	22.8 ± 9.6ab	28.5 ± 8.5b	17.0 ± 3.0a	21.9 ± 8.3ab	0.0022
*Sum*	1,972 ± 151a	2,643 ± 432b	1,856 ± 251a	2,544 ± 302b	<0.0001
**Other volatiles**					
2-Butyltetrahydro-furan	9.1 ± 1.2	8.0 ± 2.0	8.3 ± 2.1	8.1 ± 1.9	0.3807
**5-(2-Tetrahydrofurfuryl)-heptan-2-ol**	48 ± 30.3b	24.4 ± 12.7a	33.4 ± 14.3ab	23.3 ± 12.8ab	0.0024
6-Ethenyl-2,2,6-trimethyloxan-3-ol	36.3 ± 6.2c	29 ± 3.8a	34.9 ± 5.1bc	31.3 ± 4.3ab	0.0005
**Total**	5,354 ± 1,106	4,929 ± 950	4,580 ± 803	4,833 ± 619	0.1278

*Variables showing a significant interaction between treatments and phenological stages are in bold (refer to Supplementary Table 5 for repeated measure ANOVA and Tuckey's comparison test).*

a *All compounds were quantified as 2-octanol equivalents. Values are means ± standard deviation of 15 biological replicates. For each treatment, values with different letters indicate significant differences according to Tukey's test at p < 0.05. ns: not significant; nd: not determined. Repeated measure ANOVA was also carried out to detect possible interactions between temperature; treatments and phenological stages (Supplementary Table 7)*.

The total concentration of FVC did not change from EL-36 to EL-38 (Supplementary Table 4), but 12 variable compounds showed significant differences from one stage to another, mostly among aliphatic alcohols and aliphatic acids. Noticeably, methyl salicylate significantly increased from EL-36 to EL-37/EL-38, whereas benzyl alcohol significantly decreased from stage EL-36/EL-37 to stage EL-38. In contrast to the total FVC fraction, the total concentration of GVC significantly increased with ripening and was significantly higher at EL-38 with a concentration of 5.6 μg g^−1^ FW, compared to EL-36 and EL-37 with 4.4 μg·g^−1^ FW and 4.8 μg g^−1^ FW, respectively (Supplementary Table 4). The rise of total GVC is related to a significant increase in the concentration of 23 GVC, mostly aliphatic alcohols and terpenes, which totalized 181 and 1,327 ng g^−1^ FW, respectively, at EL-38. Of interest, the concentration of the monoterpenes linalool and nerol increased by 2.5 times, and the concentration of the sesquiterpene nerolidol increased by 1.3 times from stage EL-37 to EL-38.

The VC profiles also varied depending on mini-greenhouse treatment applied during the pre-veraison (Pre) or the post-veraison (PT), or during the whole season (W) (Tables 3, 4). The CT also showed different VC profiles than other treatments, especially from the Pre and W treatments. CT berries showed the highest concentration of total FVC, with 14.06 μg g^−1^ FW, compared to the W and Pre treatments, with 12.61 and 12.46 μg g^−1^ FW, respectively (Table 3). The total GVC did not vary from one treatment to another (Table 4); however, the mini-greenhouse treatments affected specific classes of compounds, and most of these changes were observed in the GVC fraction.

Pre and W treatments resulted in a higher accumulation of benzene derivatives when compared to no greenhouse (CT) and mini-greenhouse used during post-veraison (PT). Indeed, the average concentrations in the Pre and W treatments of benzyl alcohol with 1,349 ng g^−1^ FW and 2-phenylethanol with 1,138 ng g^−1^ FW were significantly higher compared to the average concentrations of benzyl alcohol of 1,049 ng g^−1^ FW and 2-phenylethanol of 763 ng g^−1^ FW found in the CT and PT (Table 4). Conversely, CT berries had higher concentrations of bound volatile phenols at 554 ng g^−1^ FW when compared to Pre and W treatments at 371 and 354 ng g^−1^ FW, respectively, comprising isoeugenol, isovanillyl alcohol, acetovanillone, methyl-3-hydroxybenzoate, (*E*)-coniferyl alcohol, and sinapyl alcohol. CT berries also contained significantly higher concentrations of terpenes at 1,322 ng g^−1^ FW and C_13_-norisoprenoids at 1,126 ng g^−1^ FW when compared to Pre and W treatments at 714 and 904 ng g^−1^ FW for terpenes and C_13_-norisoprenoids, respectively, comprising (*E*)-linalool oxide, (*E*)-8-hydroxylinalool, nerolidol, β-ionol, and 3-oxo-7,8-dihydro-α-ionol.

The use of mini-greenhouses during the post-veraison (PT) phase had a limited impact on the FVC and GVC profiles of berries but, interestingly, berries from the PT treatment showed VC profiles somewhat intermediate between the Pre and W treatments, and the CT. Besides the similar accumulation of benzene derivatives (as mentioned earlier), PT and CT berries also had a similar concentration of the terpenes nerol and nerolidol. On the other side, CT berries had significantly higher (*E*)-8-hydroxylinalool concentrations of 101 ng g^−1^ FW and β-ionol concentrations of 236 ng g^−1^ FW when compared to PT berries at 77 and 174 ng g^−1^ FW, respectively.

Certain compounds showed a significant interaction between phenological stages and the mini-greenhouse treatments, meaning that treatment impact varied according to the phenological stage (refer to Supplementary Tables 6, 7 for detailed statistics of interactions). Among FVC, the 1-hexanol concentration of 471 ng g^−1^ FW was significantly higher in CT berries when compared to 253 ng g^−1^ FW of W berries at stage EL-36, but no differences were observed between treatments at other stages (Supplementary Table 6). Conversely, bound 1-hexanol was more concentrated in PT berries at 53.6 ng g^−1^ FW when compared to W berries at 20.4 ng g^−1^ FW (Supplementary Table 7). The accumulation of bound (*Z*)-linalool oxide, hotrienol, (*Z*)-8-hydroxylinalool, and lilac alcohol C was significantly modulated by grapevine phenology and treatments. Globally, these compounds accumulated at a faster rate in CT berries and were significantly more concentrated in CT berries at stages EL-37 and/or EL-38 when compared to other treatments. The volatile phenol methyl vanillate showed a similar accumulation pattern and was significantly more concentrated in CT berries at EL-38 when compared to other treatments and phenological stages.

### Principal Component Analysis of Volatile Compounds

**Figure 2:**
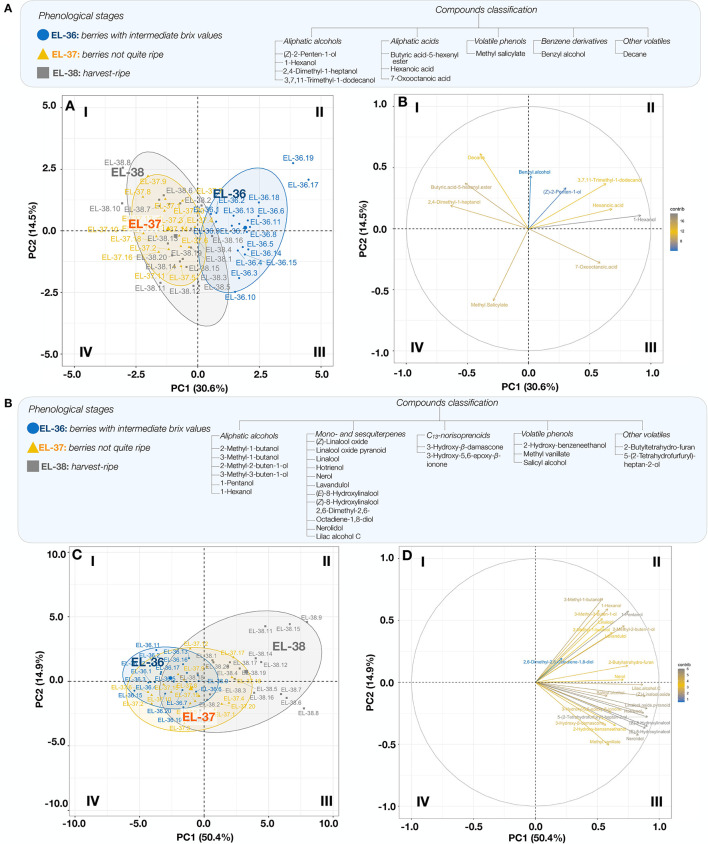
Principal component analysis (PCA) of free volatile compounds (FVCs) **(A)** and glycosylated volatile compounds (GVCs) **(B)** (compounds; right plot) at phenological stages (EL-36, EL-37, and EL-38; samples; left plot). Biological replicates are labeled 1 to 20. FVCs include the following variables: (*Z*)-2-penten-1-ol, 1-hexanol, 2,4-dimethyl-1-heptanol, 3,7,11-trimethyl-1-dodecanol, butyric acid-5-hexenyl ester, hexanoic acid, 7-oxooctanoic acid, methyl salicylate, benzyl alcohol, and decane. GVCs include the following variables : 2-methyl-1-butanol, 3-methyl-1-butanol, 2-methyl-2-buten-1-ol, 3-methyl-3-buten-1-ol, 1-pentanol, 1-hexanol, (*Z*)-linalool oxide, linalool oxide pyranoid, linalool, hotrienol, nerol, lavandulol, (*E*)-8-hydroxylinalool, (*Z*)-8-hydroxylinalool, 2,6-dimethyl-2,6-octadiene-1,8-diol, nerolidol, lilac alcohol, 3-hydroxy-β-damascone, 3-hydroxy-5,6-epoxy-β-ionone, 2-hydroxy-benzeneethanol, methyl vanillate, salicyl alcohol, 2-butyltetrahydro-furan, and 5-(2-tetrahydrofurfuryl)-heptan-2-ol. Quadrants are identified I-IV (clockwise).

Principal component analyses were performed to show the relationships between VCs and the mapping of phenological stages (Figure 2). The matrix used for this analysis consisted of FVC and GVC that varied significantly from one ripening stage to another (EL-36, EL-37, and EL-38). PC1 and PC2 of the compounds from the FVC fraction explained 45.1% of the total variability (Figure 2A). Based on this PCA, the aliphatic alcohol 1-hexanol was the main contributor to the FVC (PC 1) and was mainly associated with the ripening stage EL-36, while 2,4-dimethyl-1-heptanol, butanoic acid-5-hexenyl ester, decane, and methyl salicylate were associated with the ripening stages EL-37 and EL-38, which were less discriminated from each other. In the PCA of the GVC (Figure 2B), PC1 and PC2 explained 65.3% of the total variability between samples (PC 1, 50.4%; PC 2, 14.9%). The compounds used in this PCA were mostly associated with the phenological stage EL-38 (quadrants II and III, Figure 2B), but the terpenes (*E*)- and (*Z*)-8-hydroxylinalool, hotrienol, (*Z*)-linalool oxide, and nerolidol and lilac alcohol C were the most significant contributors to the mapping, mostly over PC 1. Groups (EL-36, EL-37, and EL-38) were better discriminated with regard to GVC when compared to FVC.

**Figure 3:**
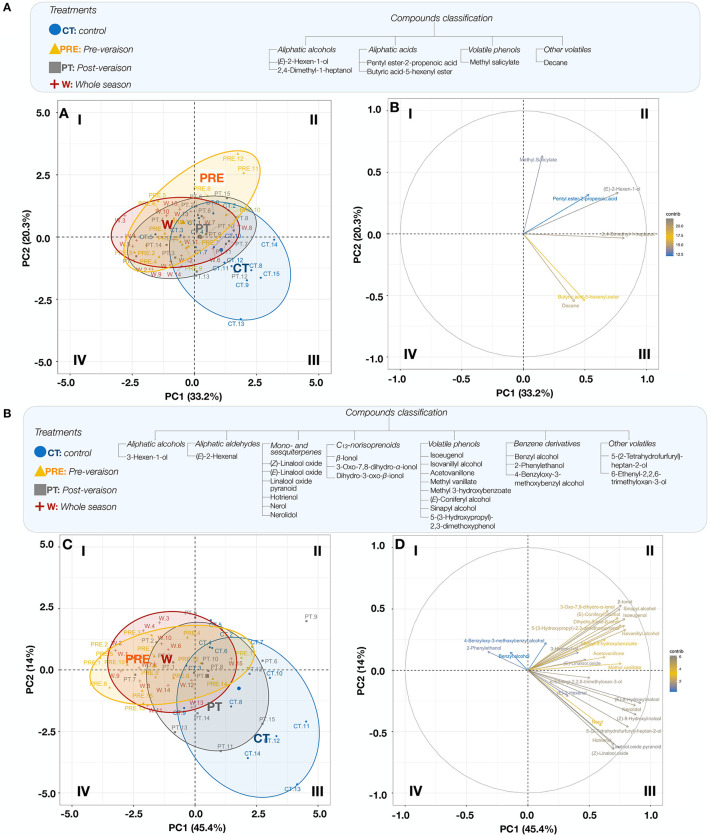
PCA of FVCs **(A)** and GVCs **(B)** (variables; right plot), plotted according to the treatments (CT, control; PRE, pre-veraison; PT, post-veraison; W, whole season; samples; left plot). Biological replicates are labeled 1 to 15. FVCs include the following variables: (*E*)-2-hexen-1-ol, 2,4-dimethyl-1-heptanol, pentyl ester-2-propenoic acid, butyric acid-5-hexenyl ester, methyl salicylate and decane. GVCs include the following variables 3-hexen-1-ol, (E)-2-hexenal, (Z)-linalool oxide, (*E*)-linalool oxide, linalool oxide pyranoid, hotrienol, nerol, nerolidol, β-ionol, 3-Oxo-7,8-dihydro-α-ionol, dihydro-3-oxo-β-ionol, isoeugenol, isovanillyl alcohol, acetovanillone, methyl vanillate, methyl 3-hydroxybenzoate, (*E*)-coniferyl alcohol, sinapyl alcohol, 5-(3-hydroxypropyl)-2,3-dimethoxyphenol, benzyl alcohol, 2-phenylethanol, 4-benzyloxy-3-methoxybenzyl alcohol, 5-(2-tetrahydrofurfuryl)-heptan-2-ol, and 6-ethenyl-2,2,6-trimethyloxan-3-ol. Quadrants are identified I-IV (clockwise).

The PCAs of FVC and GVC and temperature treatments were also performed only using significant variables (Figure 3). The PCA of FVC (Figure 3A) explained 53.5% of the variability between treatments. The aliphatic alcohols *(E)*-2-hexen-1-ol and 2,4-dimethyl-1-heptanol were the main contributors to PC 1 and mainly associated with the CT, whereas the volatile phenol methyl salicylate, the main contributor to PC 2, was associated with the treatment Pre. The PCA of GVC showed that the variability between samples was explained at 45.4% by PC 1 and at 14.0% by PC 2 (Figure 3B). The mapping of compounds and samples showed two different patterns. The first pattern showed that the aliphatic alcohol 5-(2-tetrahydrofurfuryl)-heptan-2-ol, the aliphatic aldehyde (*E*)-2-hexenal, and the monoterpenes (*Z*)-linalool oxide, hotrienol, nerolidol, and pyranoid linalool oxide were mainly associated with the CT. The second pattern showed that benzyl alcohol and 2-phenylethanol were strongly associated with the treatments W and Pre (quadrant A-I, Figure 3B). Overall, the mapping showed that treatments Pre and W were quite similar in terms of GVC profile, whereas PT and CT showed some similarities, while not being totally alike.

## Discussion

Climate change will likely modify viticulture practices and further impact wine properties in the near future (van Leeuwen and Darriet, 2016). In northern areas such as Eastern Canada, temperatures have been predicted to increase noticeably (Vasseur and Catto, 2008) but, as longer growing seasons happen more often, larger variations in temperature patterns during the season could make it even more challenging to properly ripen berries year after year. In this study, the impact of different temperature patterns on berry composition was explored at three harvest stages using on-the-row mini-greenhouses at different times during the season. The mini-greenhouses efficiently modulated temperature during the season by creating warmer conditions during the first phase of berry development (Pre treatment) or during veraison (PT treatment) while keeping a similar amount of total GDD during the season, between the treatments Pre and PT and the CT (Figure 2). The treatment lasting throughout the season (Whole) increased GDD accumulation, mimicking a temperature rise that could be expected from global warming. Both the phenological ripening stage and the mini-greenhouse treatment affected the berry biochemistry and the accumulation of VCs in *Vitis* sp. L'Acadie blanc berries.

### Berry Basic Composition

Grapevines under the mini-greenhouses throughout the whole growing season (W treatment) resulted in berries with higher TSS than the control and there was a strong positive correlation of GDD with TSS but not TA of pH (Supplementary Table 3 and Supplementary Figure 2). Elevated temperatures can impact berry biochemical composition by altering grapevine physiology and metabolism (Keller, 2015). Several studies that have previously evaluated the effects of thermal stress on berries indicate the concentration of primary metabolites and physicochemical properties vary due to the duration, intensity, and moment of exposure during development (Soar et al., 2009; Greer and Weston, 2010; Greer and Weedon, 2013; Crespo et al., 2017; Pastore et al., 2017). A positive impact of temperature on TSS was reported in Muscat à petits grain blancs and Sangiovese (Crespo et al., 2017; Pastore et al., 2017). These authors also observed a significant impact on TA and pH, whereas others (Sadras and Soar, 2009) observed no alteration of TA nor pH at a higher temperature, suggesting that other factors could be involved in organic acid metabolism in response to temperature. Several studies (Sadras and Soar, 2009; Soar et al., 2009; Greer and Weedon, 2013; Sadras et al., 2013; Pastore et al., 2017) suggested that differences in parameters, such as berry growth, TSS, pH, and TA, can respond differently depending on the duration and intensity of temperature increases and interactions with other factors (solar radiation, UV, water, and nutrient supply) and, more importantly, that the response is cultivar-dependent.

### Volatile Compounds

A total of 99 VCs were quantified in the free and glycosylated fractions of L'Acadie blanc berries. Approximately 90% of the total FVC was composed of two compounds biosynthesized from the lipoxygenase pathway, namely, ~70% of the FVC being (*E*)-2-hexenal (derived from α-linolenic acid; C18:3Δ9,12,15) and ~19% of the FVC being hexanal (derived from linoleic acid; C18:2Δ9,12) (Moreno and Peinado, 2012; Lin et al., 2019). The higher concentrations of (*E*)-2-hexenal over hexanal agree with previous studies on *Vitis vinifera* and interspecific hybrids that suggested that the C18:3 route dominates over the C18:2 route, as α-linolenic acid is the preferred substrate for the lipoxygenase enzyme (Kalua and Boss, 2009; Pedneault et al., 2013).

The distribution of the GVCs was spread across eight different chemical families, but benzene derivatives and monoterpenes represented ~37 and ~25% of the total GVC fraction, respectively. 2-Phenylethanol and benzyl alcohol represented the largest proportion of benzene derivatives (~95%). High levels of 2-phenylethanol are consistent with results from other interspecific *Vitis* sp. varieties, as 2-phenylacetaldehyde and 2-phenylethanol from the juice of “St. Croix” and “Sabrevois” studied by Slegers et al. (2015) represented 51.2% and 37.7% of the total free aroma profile, respectively. Similarly, Ghaste et al. (2015) showed that varieties, such as Isabella (*V. vinifera* × *Vitis labrusca*), *Vitis arizonica texas*, and *Vitis cinerea*, also have high levels of compounds, such as 2-phenylethanol and benzyl alcohol in the GVC fraction with concentrations reaching up to of 9,420 and 3,710 μg kg^−1^, respectively.

### Influence of the Phenological Ripening Stage on Volatile Compounds

The phenological ripening stage significantly affected the accumulation of VCs in L'Acadie blanc berries, as the concentration of most compounds increased as berries reached maturity.

Aliphatic acids from the FVC fraction, aliphatic alcohols, and monoterpenes from the GVC fraction represented the main chemical families significantly impacted by the ripening stage, the majority of which increased in the later ripening stages (Supplementary Tables 4, 5). These results agree with the literature, as many of these compounds are known to be synthesized during the last stages of berry development (Moreno and Peinado, 2012; Crespo et al., 2017). However, although the presence of VC increased with ripening, their accumulation is not necessarily uniform (Pedneault et al., 2013). For example, linalool was found at phenological ripening stages EL-37 and EL-38, but not at stage EL-36 (Supplementary Table 5). In contrast, the concentration of *(Z)*-8-hydroxylinalool, which constituted a large proportion of the GVC profile (~67% of the total monoterpenes), increased as maturity was reached (Figure 2B).

Globally, when comparing the FVC with GVC, FVC represented a larger proportion of VCs in L'Acadie blanc berries. GVC is highly soluble in aqueous media (polar compounds), which facilitates plant transport through the phloem (Moreno and Peinado, 2012). In wine, GVC can represent the potential aroma that is revealed by acid or enzymatic hydrolysis during winemaking (Dunlevy et al., 2009). Interestingly, monoterpenes were only found as glycosides in L'Acadie blanc berries. Among these, (*E*)-8-hydroxylinalool (a highly oxidized monoterpene) can undergo several oxidation and cyclization processes during the winemaking process and, ultimately, result in the production of wine lactone, an important contributor to wine aroma (Lin et al., 2019); thus, the contribution of glycosides to the wine aroma goes beyond a restricted definition of varietal aroma compounds.

### Grapevine Plasticity in Response to Temperature Increase

Besides genotype specificities, the accumulation of VCs can be influenced by seasonal and regional climates and vine-growing practices and factors that can influence the characteristics of a given variety grown in different areas (Dunlevy et al., 2009; Moreno and Peinado, 2012). In this study, 99 VCs were quantified in the free and glycosylated fractions (FVC and GVC, respectively), and their concentration was influenced by the type of treatment they were exposed to (Tables 3, 4). Besides a limited number of compounds showing significant differences [e.g., (*E*)-2-hexen-1-ol, 2,4-dimethyl-1-heptanol, 2-propenoic acid pentyl ester, and decane], the FVC profile of the berries was relatively stable across treatments. Conversely, large differences were observed between the GVC profiles of berries across different classes of compounds, including benzyl and phenolic derivatives, terpenes, and C_13_-norisprenoids.

Benzyl alcohol (~22%) and 2-phenylethanol (~18%) were the main GVC found in berries for all treatments, but their accumulation patterns differed between temperature treatments. Berries from the treatments exposed to elevated temperatures during the early developmental stages (pre-veraison phase; W and Pre treatments) had the highest concentrations of benzyl alcohol and 2-phenylethanol when compared to those exposed to higher temperature only during veraison (PT treatment) or not exposed (CT treatment; Figure 3B and Table 4). In contrast, the hydroxy-methoxy-substituted volatile phenols, such as isoeugenol, isovanillyl alcohol, acetovanillone, methyl-3-hydroxy benzoate, and (*E*)-coniferyl alcohol, had reduced accumulation in the Pre and W treatments when compared to the CT treatment. These findings suggest that mini-greenhouse treatments selectively modulated the metabolic pathways related to the biosynthesis of benzyl and phenyl derivatives at early developmental stages. 2-Phenylethanol is biosynthesized through the L-phenylalanine pathway *via* phenylpyruvic acid and phenylacetaldehyde, whereas both benzyl alcohol and hydroxy-methoxy-substituted volatile phenols derive from the phenylpropanoid pathway. Benzyl alcohol synthesis occurs during the early steps of the phenylpropanoid pathway, through the shortening by two carbons of the side chain of *trans*-cinnamic acid, while hydroxy-methoxy-substituted volatile phenols are produced later downstream, from caffeic, ferulic, and sinapic acids (Huang et al., 2001; Widhalm and Dudareva, 2015; Shu et al., 2021).

The bioconversion of L-phenylalanine into 2-phenylethanol has been found to be favored at high temperatures (Huang et al., 2001; Shu et al., 2021), but different species of plants tend to have different accumulation patterns of the compound. Zeng et al. (2019, 2020) showed an increase in the 2-phenylethanol concentration of tea (*Camellia sinensis*) leaves and cut roses (*Rosa hybrida*), following a temperature increase. In petunias (*Petunia* × hybrida cv. Mitchel Diplod), high temperatures reduced the levels of 2-phenylethanol, whereas no change was observed in tomato (*Solanum lycopersicum*) (Zeng et al., 2019). Phenylpropanoid derivatives, such as benzyl alcohol and hydroxy-methoxy-substituted volatile phenols, are also known to have different patterns of accumulation in plants, in part because they are thought to be produced on-demand in response to abiotic and biotic stress (Widhalm and Dudareva, 2015). For instance, benzyl alcohol has been shown to increase in cut roses following temperature treatment but was found in lower concentrations in Albarino grapes grown on a warm site when compared to a cooler site (Vilanova et al., 2007; Zeng et al., 2020). However, the biosynthesis of complex phenylpropanoid derivatives, such as flavonoids, produced further down the phenylpropanoid pathway, can be reduced at high temperatures, resulting in the accumulation of the benzyl alcohol precursor cinnamic acid (Austen et al., 2019). Thus, the differential accumulation of phenylpropanoid derivatives in the CT treatment (more hydroxy-methoxy-substituted volatile phenols) when compared to the Pre and W treatments (more benzyl alcohol) could be attributable to the treatment-specific differences in abiotic conditions, with the observed temperature increases potentially resulting in increased production of benzyl alcohol at the expense of other compounds in the phenylpropanoid pathway. Additional targeted analyses of phenolic compounds within the pathway are necessary to test this hypothesis; however, the red coloration (likely attributable to anthocyanins) observed on the CT berries when compared to the greener berries of W and Pre treatments (reduced anthocyanins) supports this potential impact of treatments upon the phenylpropanoid pathway (Supplementary Figure 3). From a wine perspective, such variation in phenol profiles may affect wine sensory properties and suggests that climate change could have important potential impacts on wine quality.

Terpenes contributed significantly to the glycosidic fraction (~23%) and were significantly affected by the treatments. In contrast to volatile phenols, the terpenes, such as (*Z*)-linalool oxide, (*E*)-linalool oxide, hotrienol, (*Z*)-8-hydroxylinalool, and (*E*)-8-hydroxylinalool, predominantly accumulated in the CT (Figure 3B and Table 4). As previous research (Goh et al., 2016) has shown a close positive relationship between temperature and terpene production in plants, which is dependent on the geographic origin and genotype of plant populations, this finding was unexpected. Indeed, the W treatment accumulated more GDD due to an overall higher temperature than all other treatments but had the lowest levels of monoterpenes. One explanation for decreased terpenes could be the polycarbonate panels used in on-the-row mini-greenhouses, which block UV rays below 380 nm in addition to raising the canopy temperature. Polycarbonate panels with high visible light transmission and high UV absorbance have traditionally been used in order to support plant growth while protecting equipment from long-term UV damage, but research has highlighted the interactions of UV light with plant physiology. One such interaction is thought to be with terpene biosynthesis, which is typically associated with veraison in grapevine ripening and involves, among others, a family of proteins called terpene synthases (Matarese et al., 2013). The terpene synthase family includes several genes in grapevine (up to 25 for the terpene synthase-b subfamily), and some of these genes are thought to be UV responsive and differentially expressed at different developmental stages (Carbonell-Bejerano et al., 2014; Wen et al., 2015). Differential UV exposure has been directly shown to affect the accumulation of compounds, such as C_13_-norisoprenoids and monoterpenes (Joubert et al., 2016; Young et al., 2016), generating changes in the VC profile by activating the various genes responsible for their synthesis (Lin et al., 2019). Exposure is thought to increase the production of several compounds (e.g., monoterpenes, aldehydes, alcohols, norisoprenoids, and flavonols) in order to protect the berries against UV damage (Gil et al., 2013; Joubert et al., 2016). Specifically, in grapevine, increases in monoterpenes, such as limonene and geraniol in *V. vinifera* cv Malbec (Gil et al., 2013); hotrienol, limonene, and linalool in Sauvignon blanc (Joubert et al., 2016); and geraniol, citronellol, and nerol from Pinot noir (Song et al., 2015) have been associated with UV exposure. Similar to terpenes, C_13_-norisoprenoids, including 3-oxo-α-ionol, β-ionol, and 3-oxo-7,8-dihydro-α-ionol, also accumulated in the CT treatment. Previous findings demonstrated that norisoprenoid precursors, such as β-carotene, may increase in berries exposed to UV radiation, which could in turn lead to higher C_13_-norisoprenoid concentrations (Joubert et al., 2016).

## Conclusion

This study explored the impact of mini-greenhouse treatments, installed in the vineyard at different times during the growing season, on the FVC and GVC profiles during the ripening of L'Acadie blanc berries cultivated in Nova Scotia, Canada. Mini-greenhouse treatments, even when applied only during early berry development, affected berry composition at harvest. Of interest, berries from the treatments with an increased accumulation of GDD during the first developmental stages (W and Pre) had the highest concentrations of benzyl alcohol and 2-phenylethanol, suggesting that early exposure to higher temperatures could potentially impact berry VC composition. In contrast, terpenes, C_13_-norisoprenoids, and hydroxy-methoxy-substituted volatile phenols predominantly accumulated in the CTs, possibly as an effect of reduced UV radiation due to the polycarbonate panels used in mini-greenhouses. Although plants were exposed to natural light during veraison, grapevines under the mini-greenhouses during early berry development (Pre treatment) had reduced levels of terpenes and C_13_-norisoprenoids compared to CTs, suggesting UV exposure might be more important during berry development as well as temperature.

Despite the study only being conducted over a single growing season, these findings provide insight into the biochemical plasticity of *Vitis* sp. and the possibility of modifying the accumulation of VCs in berries. 2-Phenylethanol and benzyl alcohol were the main varietal aroma compounds found in L'Acadie blanc berries and were increased in vines with higher GDD, suggesting that increased temperatures attributable to climate change could potentially affect the quality of L'Acadie blanc wines in the future. Future research would need to establish the consistency of this pattern in field trials without differential UV exposure to confirm this possibility.

## Data Availability Statement

The raw data supporting the conclusions of this article will be made available by the authors, without undue reservation.

## Author Contributions

KP conceived, planned this study, and supervised FC-A and GS. KP, NB, and FP contributed to the planning of the experiment and the realization in the field. FC-A and GS implemented and maintained the viticultural treatments and carried out berry sampling. FC-A did the processing and analysis of climatic data and berry samples. PN developed the method for FVC and GVCs extraction. FC-A and KP drafted the original manuscript and finalized the manuscript. MD, NB, and FP reviewed and edited the manuscript. All authors approved the manuscript for publication.

## Funding

This research was made possible by the financial support of Agriculture and Agri-Food Canada (AAFC), the Nova Scotia Department of Agriculture, the Canadian Grapevine Certification Network (CGCN), the Grape Growers' Association of Nova Scotia, through Canadian Agricultural Partnership's AgriScience Clusters (Activity 9B), and the Natural Sciences and Engineering Research Council of Canada (NSERC Discovery grant).

## Conflict of Interest

The authors declare that the research was conducted in the absence of any commercial or financial relationships that could be construed as a potential conflict of interest.

## Publisher's Note

All claims expressed in this article are solely those of the authors and do not necessarily represent those of their affiliated organizations, or those of the publisher, the editors and the reviewers. Any product that may be evaluated in this article, or claim that may be made by its manufacturer, is not guaranteed or endorsed by the publisher.
