# Supplementary material for: Different Temperature and UV Patterns Modulate Berry Maturation and Volatile Compounds Accumulation in Vitis sp
Source: Front Plant Sci. 2022 Jun 30;13:862259. doi: 10.3389/fpls.2022.862259 (PMC9280473; doi:10.3389/fpls.2022.862259)
Supplement: Supplementary file 1 [file Data_Sheet_1.PDF]

Supplementary Material

1 Supplementary Tables

Supplementary table 1. Authentic standards used to determine the retention time of 20 of the total compounds found in this study.

Nomenclature	CAS[a] No.	Formula	Supplier	Purity	Retention time
Benzaldehyde	100-52-7	C_7H_6O	Fluka	≥ 99 %	11.79
Hexanoic acid	142-62-1	$C_6H_{12}O_2$	Sigma-Aldrich	≤ 100 %	12.81
2-octanol	123-96-6	$C_8H_{18}O$	Sigma-Aldrich	≥ 99.5 %	13.34
(E,E)-2,4-Heptadienal	4313,03,5	$C_7H_{10}O$	SAFC	≥ 88 %	13.63
2-Ethyl-1-hexanol	104-76-7	$C_8H_{18}O$	Sigma-Aldrich	≥ 99.6 %	14.41
Benzyl alcohol	100-51-6	C_7H_8O	Sigma-Aldrich	100 %	14.60
(Z)-Linalool oxide	60047-17-8	$C_{10}H_{18}O_2$	Sigma-Aldrich	≥ 97 %	16.10
Linalool	78-70-6	$C_{10}H_{18}O$	Sigma-Aldrich	97 %	17.19
2-phenylethanol	60-12-8	$C_8H_{10}O$	Sigma-Aldrich	≥ 99 %	17.70
Methyl salicylate	119-36-8	$C_8H_8O_3$	Sigma-Aldrich	≥ 98 %	20.91
4-Allylanisole	140-67-0	$C_{10}H_{12}O$	Sigma-Aldrich	98 %	21.07
Decanal	112-31-2	$C_{10}H_{20}O$	Sigma-Aldrich	≥ 98 %	21.31
Nerol	106-25-2	$C_{10}H_{18}O$	SAFC	97 %	22.25
Eugenol	97-53-0	$C_{10}H_{12}O_2$	Sigma-Aldrich	≥ 98 %	27.08
α-ionol	25312-34-9	$C_{13}H_{22}O$	Sigma-Aldrich	≥ 90 %	27.84
α-ionone	127-41-3	$C_{13}H_{20}O$	Sigma-Aldrich	≥ 90 %	29.60
Isoeugenol	97-54-1	$C_{10}H_{12}O_2$	Sigma-Aldrich	98 %	30.35
β-ionone	79-77-6	$C_{13}H_{20}O$	Sigma-Aldrich	≥ 95 %	31.63
2,4-di-tert-butylphenol	96-76-4	$C_{14}H_{22}O$	Sigma-Aldrich	99 %	32.49
Ethyl vanillate	617-05-0	$C_{10}H_{12}O_4$	Sigma-Aldrich	≤ 100 %	34.98

Abbreviations: [a]CAS: Chemical Abstracts Service.

Supplementary table 2. Productivity parameters of L'Acadie blanc grapevine under the effect of different treatments for vintage 2020.

Variables[a]	CT				Pre				Post				W				p-value
Clusters per vine	26.15	±	6.42		24.00	±	8.02		21.47	±	4.90		25.28	±	6.35		0.14
Cluster wight at maturity (g)	91.87	±	32.94	*ab*	85.11	±	24.02	*a*	112.72	±	34.96	*b*	73.91	±	22.86	*a*	0.0014
Production (kg $vine^{-1}$)	2.44	±	1.04		1.99	±	0.67		2.44	±	1.06		1.86	±	0.81		0.118

[a]Data are means ± standard deviation. n=20 for CT, 18 for Pre and W and 19 for Post. For each variable values with different letters indicate significant differences between treatments according to Tukey's test at p<0.05.

Supplementary table 3. Pearson correlations among environmental variables (GDD) and berry maturity variables (Berry weight (100 berries), TSS, TA and pH) in Vitis sp. L'Acadie blanc. ($52 \leq n \leq 58$).

Correlated variables[a]	Correlation coefficient (r)	r-squared	Significance (*p*)
Berry weight (100 berries) vs. TSS	0.25	0.0625	0.291
Berry weight (100 berries) vs. pH	0.4	0.16	0.014*
Berry weight (100 berries) vs. TA	-0.32	0.1024	0.095
Berry weight (100 berries) vs. GDD	2.13E-03	4.5369E-06	> .999
TSS vs. pH	0.66	0.4356	< .001***
TSS vs. TA	-0.78	0.6084	< .001***
TSS vs. GDD	0.7	0.49	< .001***
pH vs. TA	-0.77	0.5929	< .001***
pH vs. GDD	0.19	0.0361	0.497
TA vs. GDD	-0.36	0.1296	0.061

[a]For each correlation values with an asterisk (*) indicate significant correlation between parameters at p<0.05 and with three asterisk (***) significant at p<0.001.

Supplementary table 4. Impact of maturity stages (EL-36, EL-37 and EL-38) on the profile of free volatile compounds (ng · g^{-1} FW) of berries from *Vitis* sp. cv. L'Acadie blanc in 2020. Variables showing significant interaction between treatments and phenological stages are in bold; see Supplemental table 7 for Tuckey's comparison test).

Compounds[a]	EL-36	EL-37	EL-38	p-value
Aliphatic alcohols				
(Z)-2-Penten-1-ol	40.3 ± 11.8 b	35.4 ± 12.1 ab	30.2 ± 10.5 a	0.0333
**1-Hexanol**	348 ± 125 b	33.8 ± 30.7 a	30.7 ± 30.2 a	<.0001
2-Hexanol	3.2 ± 2.3	3.0 ± 1.8	2.7 ± 1.7	0.8470
(E)-2-Hexen-1-ol	296 ± 148	374 ± 114.7	375 ± 132	0.1050
2,4-Dimethyl-1-heptanol	11.5 ± 6.2 a	354 ± 159 b	365 ± 159 b	<.0001
2,6-Dimethyl-2-octanol	12.3 ± 9.2	15.3 ± 10.3	12.9 ± 11.2	0.6800
3,7,11-Trimethyl-1-dodecanol	35.6 ± 33.8 b	7.9 ± 5.5 a	8.5 ± 4.2 a	0.0001
Sum	735 ± 270	817 ± 244	813 ± 264	0.5320
Alpihatic aldehydes				
Hexanal	2 420 ± 392	2 332 ± 459	2 549 ± 357	0.2410
2-Hexenal	77.7 ± 15	79.3 ± 18.0	90.2 ± 20.4	0.0642
(E)-2-Hexenal	9 547 ± 1 096	8 998 ± 1 404	9 055 ± 880	0.2590
2,3,4-Trimethyl-hex-3-enal	2.9 ± nd	2.5 ± 0.5	26.7 ± 51.8	0.7860
(E,E)-2,4-Hexadienal	3.2 ± 0.8	2.5 ± 0.9	3.3 ± 1.0	0.0588
Nonanal	10 ± 7.1	9.4 ± 7.8	10.2 ± 6.6	0.9370
(E,E)-2,6-Nonadienal	12.5 ± 4	12.2 ± 5.2	10.6 ± 2.6	0.2710
(E)-4-Undecenal	1.6 ± 0.7	1.6 ± 1.2	1.7 ± 0.6	0.8930
Sum	12 069 ± 1 462	11 435 ± 1 848	11 727 ± 1 176	0.4224
Aliphatic acids				
2-Propenoic acid pentyl ester	75.6 ± 34.4	76.5 ± 30.8	85.2 ± 38	0.6290
**Butanoic acid-5-hexenyl ester**	6.3 ± 2.1 a	9.1 ± 2.7 ab	10.3 ± 4.5 b	0.0055
Butanoic acid octyl ester	16.1 ± 6.6	13.2 ± 7.2	15.0 ± 8.9	0.4780
Hexanoic acid	38.2 ± 14.7 b	18.4 ± 7.3 a	29.8 ± 12.6 b	<.0001
(E)-2-Hexenoic acid	20.8 ± 19.8 ab	8.7 ± 3.9 b	38.9 ± 17.4 a	0.0216
2-Ethyl-hexanoic acid	2.9 ± 1.0	2.7 ± 2.0	2.7 ± 1.1	0.9810

Heptanoic acid	5.0 ± 2.7	3.9 ± 1.8	3.8 ± 1.4	0.1670
Octanoic acid	12.2 ± 5.0	11.9 ± 6.2	12.9 ± 5.7	0.8260
7-Oxooctanoic acid	13.4 ± 5.9 b	9 ± 3.7 a	10.1 ± 3.8 ab	0.0132
Sum	171 ± 49 ab	144 ± 42 a	207 ± 58.4 b	0.0008
Aliphatic esters				
2-Butoxy-ethanol	4.4 ± 1.4	4 ± 1.5	4.7 ± 1.0	0.2530
2,2-Butoxyethoxy-ethanol	17.2 ± 8.1	16.0 ± 8.2	15.9 ± 7.7	0.8570
Sum	21.4 ± 8.9	19.7 ± 9	20.7 ± 8.2	0.8160
Volatile phenols				
**Methyl salicylate**	7.5 ± 4.9 a	12.8 ± 5.4 b	10.7 ± 8.3 ab	0.0361
Vanillin	26.9 ± 12.7	30.7 ± 26.6	28.1 ± 14.0	0.8060
4-Hydroxy-3,5-dimethoxy-benzaldehyde	11.8 ± 8.5	14.6 ± 14.7	17.8 ± 10.0	0.2530
Sum	46.2 ± 18.5	58.1 ± 40.7	56.6 ± 15.2	0.3280
Benzene derivatives				
Benzyl alcohol	31.5 ± 10.2 b	39.2 ± 8.9 b	22.6 ± 13.5 a	<.0001
Phenylethanal	22.0 ± 22.8	16.8 ± 18.7	20.0 ± 13.3	0.6770
p-Tolualdehyde	10.1 ± 10.2	11.9 ± 13.2	10.9 ± 13.5	0.9230
Benzophenone	12.1 ± 1.8	11.4 ± 0.9	11.8 ± 1.5	0.2890
2-Phenoxy-ethanol	5.1 ± 2.1	5.6 ± 3.0	5.8 ± 2.3	0.7450
Sum	75.7 ± 32.1	84.9 ± 27.2	68.9 ± 24.3	0.2060
Other volatiles				
3,4,4-Trimethyl-2-hexene	6.6 ± 0.8	6.5 ± 0.4	7.2 ± 0.8	0.2990
**Heptane**	45.1 ± 20.8	53.6 ± 20.1	55.1 ± 21.6	0.2690
4-Methyl-heptane	13.5 ± 10.8	12.6 ± 12.4	11.2 ± 8.8	0.8380
2,4-Dimethyl-heptane	5.8 ± 4.3	5.7 ± 5.6	4.8 ± 3.6	0.8240
2,4-Dimethyl-1-heptene	36.6 ± 14	39.4 ± 33	31.9 ± 22.3	0.8440
4-Propyl-3-heptene	2.3 ± 0.3	2.1 ± 0.3	2.5 ± 0.7	0.6950
2, 3, 3-Trimethyl-1,7-octadiene	5.7 ± 4.4	3.0 ± 0.4	2.0 ± 0.8	0.1290
2,2-Dimethyl-3-octene	5.5 ± 2.4	5.1 ± 2.2	5.7 ± 2.6	0.7330
Decane	9.3 ± 9.2 a	24.2 ± 9.9 b	23.1 ± 12.9 b	0.0192

4-Ethyl-decane	2.9 ± 1.8	3.8 ± 2.1	4.4 ± 3.1	0.3080
y-Undecalactone	4.9 ± 3.1	4 ± 5.1	2.7 ± 1.4	0.1730
Sum	94.8 ± 43	119 ± 47.5	118 ± 49.4	0.1900
Total	13 212 ± 1 584	12 677 ± 1 841	13 010 ± 1 220	0.5560

[a]All compounds were quantified as 2-octanol equivalents. Values are means ± standard deviation of 20 biological replicates. For each phenological stage, values with different letters indicate significant differences according to Tukey's test at p<0.05. ns: not significant; nd: not determined. Repeated measure ANOVA was also carried out to detect possible interactions between temperature; treatments and phenological stages (**Supplementary Table 6**).

Supplementary table 5. Impact of maturity stages (EL-36, EL-37 and EL-38) on the profile of glycosylated volatile compounds (ng · g^{-1} FW) of berries from *Vitis* sp. cv. L'Acadie blanc in 2020. Variables showing significant interaction between treatments and phenological stages are in bold; see Supplemental table 8 for Tuckey's comparison test).

Compounds[a]	EL-36	EL-37	EL-38	p-value
Aliphatic alcohols				
2-Methyl-1-butanol	19.1 ± 7 a	18.5 ± 8.9 a	27.7 ± 7.9 b	0.0007
3-Methyl-1-butanol	23.3 ± 5.7 a	21.8 ± 7.1 a	29.4 ± 6.7 b	0.0011
2-Methyl-2-buten-1-ol	10.2 ± 2.9 a	11.5 ± 3.8 a	17.3 ± 3.9 b	<.0001
3-Methyl-3-buten-1-ol	43.5 ± 7.8 a	41.4 ± 10.9 a	51.7 ± 10.2 b	0.0035
1-Pentanol	5.6 ± 1.4 a	7.1 ± 2.3 a	10.5 ± 3.7 b	<.0001
**1-Hexanol**	16.6 ± 6.9 a	19.1 ± 9.4 a	33.5 ± 21.2 b	0.0006
3-Hexen-1-ol	11.8 ± 6.1	12.8 ± 4.7	10.7 ± 6.0	0.517
Sum	130 ± 27.4 a	132 ± 37.3 a	181 ± 44.8 b	<.0001
Aliphatic aldehydes				
Hexanal	10.7 ± 5.2	10.9 ± 5.0	11.5 ± 5.8	0.8743
***(E)*-2-Hexenal**	25.3 ± 13.8	23.5 ± 11.9	34.6 ± 26.2	0.1292
Sum	36 ± 17.7	34.4 ± 15.6	46.2 ± 29.4	0.1865
Aliphatic acids				
Tetradecanoic acid	45.4 ± 18.9	45.3 ± 21.2	44 ± 19.4	0.9710
(Z)-9-octadecenoic acid	31.9 ± 14.1	31.8 ± 16.2	27.4 ± 11.2	0.5166
Sum	77.3 ± 22.8	77.1 ± 34.2	71.5 ± 23	0.7436
Mono- and sesquiterpenes				
***(Z)*-Linalool oxide**	28.4 ± 7.8 a	46.5 ± 15.1 b	63 ± 18.6 c	<.0001
(E)-Linalool oxide	31.7 ± 5.7	34.5 ± 9.9	31.3 ± 4.1	0.2847
**Linalool oxide pyranoid**	26.6 ± 9 a	39.9 ± 12.2 b	57.4 ± 16.2 c	<.0001
Linalool	nd ± nd	6.8 ± 2.7 a	16.6 ± 11.1 b	<.0001
**Hotrienol**	22.2 ± 13.5 a	61.1 ± 26.4 b	117.6 ± 59.0 c	<.0001
Nerol	9.8 ± 6.1 a	10 ± 5.3 a	25.2 ± 18.1 b	<.0001
**Lavandulol**	15.5 ± 2.5 a	15.5 ± 2.9 a	20.2 ± 6.3 b	0.0008
(E)-8-Hydroxylinalool	55.1 ± 16.1 a	70.6 ± 22.6 a	97.0 ± 32.2 b	<.0001

***(Z)*-8-Hydroxylinalool**	329 ± 151 a	528 ± 180 b	886 ± 378 c	<.0001
Linalyl isobutyrate	23.3 ± 7	28.3 ± 8.7	30.2 ± 13.5	0.0905
2,6-Dimethyl-2,6-octadiene-1,8-diol	nd ± nd	10.8 ± 7.6	12.5 ± 5.2	0.4320
Nerolidol	8.4 ± 2.3 a	11.1 ± 2.9 b	13.9 ± 4.4 c	<.0001
**Lilac alcohol C**	4 ± 1.8 a	6.6 ± 2.5 b	14.5 ± 3.6 c	<.0001
Sum	554 ± 199 a	869 ± 263 b	1 384 ± 516 c	<.0001
C_{13}-norisoprenoids				
3-Hydroxy-*β*-damascone	136 ± 37.5 a	157 ± 25 ab	167 ± 35.8 b	0.0147
3-Hydroxy-7,8-dihydro-*β*-ionol	69.2 ± 18.5	72.1 ± 16.2	81 ± 33.7	0.2765
3-Oxo-*α*-ionol	284 ± 75.2	331 ± 65.4	326 ± 65	0.0710
*β-I*onol	190 ± 65.2	180 ± 44.9	177 ± 86.8	0.8181
3-Hydroxy-5,6-epoxy-*β*-ionone	20.8 ± 4.6 a	20.9 ± 5.9 a	25.5 ± 4.9 b	0.0073
3-Oxo-7,8-dihydro-*α*-ionol	213 ± 33	212 ± 36.8	202 ± 38.6	0.5609
Dihydro-3-oxo-*β*-ionol	17.2 ± 5.4	17.9 ± 4.1	20.2 ± 7.2	0.2305
Sum	931 ± 187	991 ± 184	999 ± 258	0.5420
Volatile phenols				
p-Vinylguaiacol	19.4 ± 8.9	17 ± 9.1	19.6 ± 7.2	0.5527
Eugenol	32.8 ± 10.8	38.4 ± 15.6	40.4 ± 19	0.2772
Methoxyeugenol	10.4 ± 2.3	9.9 ± 1.9	11.4 ± 3.4	0.1847
2-Hydroxy-benzeneethanol	10.2 ± 7 a	9.6 ± 6.3 a	20.9 ± 12.3 b	0.0002
Isoeugenol	16.5 ± 8.9	13.4 ± 5.3	15.2 ± 11.2	0.5539
Isovanillyl alcohol	25.6 ± 10.2	23 ± 11.1	26.8 ± 17.1	0.6477
Acetovanillone	27.4 ± 5.3	25.3 ± 7.1	29.5 ± 4.9	0.0845
**Methyl vanillate**	59.5 ± 21.4 a	60.2 ± 19.9 a	119 ± 129 b	0.0231
Methyl 3-hydroxybenzoate	25.6 ± 11.2	25.2 ± 8.3	26.6 ± 10.7	0.9062
(E)-Coniferyl alcohol	39.5 ± 28.7	34.7 ± 28.5	40.4 ± 26.2	0.7870
Sinapyl alcohol	24.3 ± 16.9	21.4 ± 11.7	24.3 ± 22.4	0.8350
Salicyl alcohol	15.2 ± 5.8 a	17.0 ± 6.0 ab	20.9 ± 8.0 b	0.0268
5-(3-Hydroxypropyl)-2,3-dimethoxyphenol	9.2 ± 4.4	10.2 ± 6.3	11.9 ± 8.5	0.4336
2-Hydroxy-4,5-dimethylacetophenone	33 ± 8.7	34.9 ± 12.5	33.6 ± 11.5	0.8696

4-tert-Butyl-2-methylphenol	48.6 ± 11.5	49 ± 11.7	48.2 ± 12.4	0.9783
Sum	398 ± 111	389 ± 111	489 ± 244	0.1220
Benzene derivatives				
Benzyl alcohol	1 180 ± 228	1 182 ± 211	1 234 ± 305	0.7425
2-Phenylethanol	921 ± 227	941 ± 211	988 ± 256.1	0.6474
3-Tridecyl ester-m-toluic acid	90 ± 56.9	80.5 ± 23.5	76.2 ± 19.5	0.4928
4-Benzyloxy-3-methoxybenzyl alcohol	22.3 ± 8.6	22.1 ± 6.7	23.2 ± 10.5	0.9121
Sum	2 213 ± 427	2 226 ± 400	2 322 ± 540	0.7180
Other volatiles				
2-Butyltetrahydro-furan	7.1 ± 1.1 a	8.2 ± 1.5 b	9.9 ± 1.6 c	<.0001
**5-(2-Tetrahydrofurfuryl)-heptan-2-ol**	17.6 ± 7.2 a	27.5 ± 11.1 a	51.7 ± 23.5 b	<.0001
6-Ethenyl-2,2,6-trimethyloxan-3-ol	32.5 ± 5	33.4 ± 7.4	32.8 ± 4.3	0.8795
Total	4 396 ± 544 a	4 788 ± 671 a	5 587 ± 1 024 b	<.0001

[a]All compounds were quantified as 2-octanol equivalents. Values are means ± standard deviation of 20 biological replicates. For each phenological stage, values with different letters indicate significant differences according to Tukey's test at $p<0.05$. ns: not significant; nd: not determined. Repeated measure ANOVA was also carried out to detect possible interactions between temperature; treatments and phenological stages (**Supplementary Table 7**).

Supplementary table 6. The impact of temperature treatments (CT (control), PRE (pre-veraison), PT (post-veraison) and W (whole season)) on the profile of free volatile compounds (ng g^{-1}) from L'Acadie blanc berries harvested at three different phenological stages in 2020. Variables showing significant interaction between treatments and phenological stages are in bold. T: Treatment; PS: Phenological Stage. TxPS: interaction treatment x phenological stage. *nd*: not determined.

Compounds[a]	EL-36				EL-37				EL-38				*p*-value
	CT	Pre	PT	W	CT	Pre	PT	W	CT	Pre	PT	W	TxPS
Aliphatic alcohols													
(Z)-2-Penten-1-ol	45.1	41.9	40.7	33.3	45.2	37.9	33.0	22.8	34.9	22.8	26.7	35.0	0.1493
**1-Hexanol**	471 *c*	316 *bc*	351 *bc*	253 *b*	18.9 *a*	48.1 a	18.5 a	49.8 a	19.4 *a*	51.8 *a*	36.1 *a*	16.7 *a*	0.0033
2-Hexanol	4.4	1.5	2.8	4.0	2.4	3.1	3.0	3.3	3.0	1.6	5.3	2.2	*nd*
(E)-2-Hexen-1-ol	449	230	337	167	381	330	421	362	395	448	370	289	0.1060
2,4-Dimethyl-1-heptanol	15.5	10.2	13.8	7.5	440	264	431	281	544	343	370	200	0.0657
2,6-Dimethyl-2-octanol	4.2	17.9	15.6	13.0	12.8	13.4	16.7	17.2	20.0	8.3	14.4	10.9	0.4903
3,7,11-Trimethyl-1-dodecanol	62.8	20.1	25.0	30.5	10.2	5.1	9.9	5.5	9.0	7.1	11.3	6.7	0.2124
Sum	1 042	623	779	495	907	696	932	735	1 017	878	801	554	0.2826
Alpihatic aldehydes													
Hexanal	2 603	2 372	2 344	2 359	2 682	2 233	2 095	2 319	2 539	2 263	2 897	2 499	0.1411
2-Hexenal	72.1	84.0	79.7	75.2	85.0	75.5	76.1	80.5	101	90.0	85.3	84.3	0.7328
(E)-2-Hexenal	10 274	9 168	9 285	9 461	10 079	8 899	7 915	9 098	9 275	8 686	9 572	8 688	0.2920
(E,E)-2,4-Hexadienal	2.9	3.5	3.3	3.3	3.0	1.9	1.9	3.0	3.6	3.0	3.4	3.2	0.4238
2,3,4-Trimethyl-hex-3-enal	*nd*	*nd*	2.9	*nd*	2.1	2.8	*nd*	*nd*	3.3	61.4	*nd*	3.8	*nd*
(E,E)-2,6-nonadienal	11.3	14.0	13.0	11.8	10.8	14.7	12.9	10.5	10.7	11.0	11.6	9.0	0.9698

Nonanal	11.9	10.4	9.0	8.5	8.3	13.6	7.2	8.6	7.9	8.0	9.6	15.5	0.4897
(E)-4-Undecenal	1.4	1.3	2.2	1.5	1.5	2.4	1.1	1.6	1.5	1.9	1.8	1.8	0.4802
Sum	12 975	11 649	11 734	11 917	12 870	11 240	10 108	11 520	11 940	11 087	12 580	11 300	0.2666
Aliphatic acids													
2-Propenoic acid pentyl ester	113	56.5	79.4	54.2	84.0	85.8	67.7	68.3	99.2	56.5	94.7	90.2	0.2101
**Butanoic acid-5-hexenyl ester**	5.9 *a*	6.2 *ab*	8.0 *ab*	5.1 *ab*	11.5 *bc*	8.1 *ab*	8.8 *ab*	8.1 ab	15.5 *c*	7.5 *ab*	7.4 *ab*	10.7 *abc*	0.0016
Butanoic acid octyl ester	11.2	18.6	16.4	18.3	15.6	13.5	11.7	11.9	11.0	12.5	16.3	20.0	0.4666
Hexanoic acid	52.3	33.5	40.1	26.8	17.4	16.6	19.2	20.6	26.5	32.7	29.0	30.9	0.0564
(E)-2-Hexenoic acid	42.9	28.5	9.9	7.7	11.4	*nd*	*nd*	5.9	37.7	32.1	50.5	35.5	*nd*
2-Ethyl-hexanoic acid	*nd*	*nd*	2.6	3.1	3.6	1.9	1.7	2.3	2.1	3.1	3.8	2.3	*nd*
Heptanoic acid	4.5	3.6	5.6	5.7	5.5	3.7	2.6	3.8	3.2	3.3	4.4	4.1	0.3031
Octanoic acid	11.0	10.0	13.0	14.9	14.1	14.4	9.6	9.3	11.5	9.8	15.8	14.6	0.1907
Sum	220	150	177	138	160	153	128	134	213	166	230	219	0.1529
Aliphatic esters													
2-Butoxy-ethanol	3.6	4.2	4.8	4.9	4.0	4.5	3.5	4.2	4.8	4.0	4.9	5.1	0.5847
2,2-Butoxyethoxy-ethanol	12.4	19.0	13.8	23.6	17.1	16.3	12.7	18.1	13.9	15.1	16.7	18.1	0.7529
Sum	15.3	23.3	18.7	28.5	21.1	19.0	16.2	22.3	18.7	19.1	21.7	23.2	0.6872
Volatile phenols													
**Methyl salicylate**	4.9 *a*	9.2 *a*	9.8 *ab*	6.0 *a*	10.9 *ab*	14.5 *ab*	13.6 *ab*	12.2 *ab*	6.3 *a*	20.9 *b*	7.0 *a*	8.6 *ab*	0.0436
Vanillin	30.7	22.4	26.5	28.0	28.4	50.2	18.6	25.8	23.3	26.1	26.7	36.2	0.1709
4-Hydroxy-3,5-dimethoxy-benzaldehyde	14.3	7.6	12.8	12.5	15.1	21.3	13.4	8.6	21.6	16.6	17.9	15.3	0.7370

Sum	50.0	39.2	49.1	46.5	54.3	86.1	45.6	46.5	51.1	63.6	51.6	60.2	0.4311
Benzene derivatives													
Benzyl alcohol	39.4	30.0	29.7	26.8	35.0	43.9	38.1	39.8	30.6	23.4	15.0	21.7	0.2880
Phenylethanal	33.5	9.0	34.3	11.0	15.0	12.5	26.7	12.9	25.2	14.7	18.1	22.0	0.3004
p-Tolualdehyde	3.8	9.0	19.2	10.2	14.1	16.1	4.7	12.8	13.6	3.5	10.5	16.6	0.1415
Benzophenone	11.4	11.3	12.8	12.9	11.3	11.7	10.8	11.7	11.6	12.3	11.9	11.6	0.5205
2-Phenoxy-ethanol	5.0	4.9	6.0	4.7	4.7	7.4	4.5	5.8	4.5	6.0	6.4	6.1	0.5184
Sum	92.3	56.9	93.1	60.5	80.1	91.6	84.8	82.9	82.8	55.2	59.7	78.1	0.0443
Other volatiles													
3,4,4-Trimethyl-2-hexene	*nd*	6.4	6.7	6.7	6.8	6.2	6.0	*nd*	7.2	*nd*	8.2	6.4	*nd*
**Heptane**	27.1 *a*	44.8 *ab*	43.5 *ab*	64.9 *ab*	71.0 *b*	51.6 *ab*	48.0 *ab*	43.8 *ab*	54.3 *ab*	47.4 *ab*	56.1 *ab*	62.5 *ab*	0.0470
4-Methyl-heptane	11.5	8.5	23.0	9.0	16.4	11.1	7.3	14.5	10.1	6.8	19.7	11.9	0.3266
2,4-Dimethyl-heptane	2.6	4.4	9.6	5.1	12.0	4.1	2.4	5.0	4.9	3.1	7.9	4.3	0.0586
2,4-Dimethyl-1-heptene	32.7	34.5	47.8	32.5	90.1	54.6	25.4	13.4	66.2	15.2	56.4	21.4	*nd*
4-Propyl-3-heptene	*nd*	2.2	2.0	2.6	2.3	1.8	*nd*	*nd*	2.5	2.1	3.4	2.1	*nd*
2,2-Dimethyl-3-octene	3.6	5.3	6.4	6.4	6.6	4.7	5.0	4.3	7.0	4.4	6.0	5.6	0.2508
2, 3, 3-Trimethyl-1,7-octadiene	7.4	1.2	6.0	5.9	3.4	2.8	2.6	*nd*	1.6	3.4	2.0	1.4	*nd*
4-Methyl-nonane	3.6	5.2	5.9	5.5	6.8	4.8	3.8	5.6	5.6	4.4	5.2	5.1	0.1165
Decane	*nd*	2.4	4.0	12.3	28.4	20.0	24.0	24.2	28.7	18.2	32.5	12.7	*nd*
4-Ethyl-decane	2.1	3.4	4.8	2.5	3.4	4.1	2.6	5.0	4.6	3.2	6.8	3.0	0.4910
y-Undecalactone	5.5	4.7	5.7	4.4	3.2	6.7	2.8	2.5	2.8	2.1	3.1	2.8	0.7215
Sum	62.3 *a*	74.3 *ab*	112 *ab*	130 *ab*	162 *b*	111 *ab*	97.6 *ab*	106 *ab*	132 *ab*	83.7 *ab*	131 *ab*	124 *ab*	0.0265

Total	14 456	12 616	12 963	12 815		14 254	12 396	11 413	12 646		13 455	12 353	13 875	12 359		0.289 8

[a]All compounds were quantified as 2-octanol equivalents. Data are means of n=5.For each compound, values with different letters indicate significant differences according to Tukey's test at $p<0.05$, in terms of interaction. ns: not significant; nd: not determined.

Supplementary table 7. The impact of temperature treatments (CT (control), PRE (pre-veraison), PT (post-veraison) and W (whole season)) on the profile of glycosylated volatile compounds (ng g^{-1}) from L'Acadie blanc berries harvested at three different phenological stages in 2020. Variables showing significant interaction between treatments and phenological stages are in bold. T: Treatment; PS: Phenological Stage. TxPS: interaction treatment x phenological stage. *nd*: not determined.

Compounds	EL-36				EL-37				EL-38				*p*-value
	CT	Pre	PT	W	CT	Pre	PT	W	CT	Pre	PT	W	TxPS
Aliphatic alcohols													
2-Methyl-1-butanol	25.5	17.5	19.1	14.4	18.6	21.3	13.7	20.4	29.2	33.8	24.2	23.8	0.2856
3-Methyl-1-butanol	24.9	24.7	21.3	22.4	25.6	21.2	15.5	25.0	29.7	30.8	31.4	25.8	0.2330
2-Methyl-2-buten-1-ol	9.3	12.8	9.5	9.4	12.3	10.8	11.4	11.6	18.5	16.7	18.3	15.8	0.3599
3-Methyl-3-buten-1-ol	46.8	46.4	41.9	39.0	44.8	41.8	31.1	48.1	54.0	51.2	50.2	51.4	0.3280
1-Pentanol	6.1	6.4	5.5	4.4	8.9	6.9	5.6	7.1	12.0	10.1	12.2	7.6	0.1655
3-Hexen-1-ol	17.8	8.2	11.7	9.5	14.6	9.6	13.5	13.4	13.2	7.0	16.1	6.5	0.2495
**1-Hexanol**	15.5 *a*	19.5 *a*	17.8 *a*	13.5 *a*	25.9 *a*	15.8 *a*	15.5 *a*	19.2 *a*	34.6 *ab*	25.4 *ab*	53.6 *b*	20.4 *a*	0.0309
Sum	146	136	127	113	151	127	106	145	191	175	206	151	0.1977
Aliphatic aldehydes													
Hexanal	12.7	13.4	10.1	6.6	9.3	8.6	15.7	9.9	13.4	11.3	12.1	9.3	0.2107
***(E)*-2-Hexenal**	32.6 *ab*	29.6 *ab*	23.0 *a*	16.0 *a*	23.0 *ab*	19.3 *ab*	28.1 *ab*	23.5 *ab*	40.6 *ab*	20.3 *ab*	58.7 *b*	18.9 *ab*	0.0391
Sum	45.3	43.0	33.1	22.7	32.3	28.0	43.8	33.4	54.1	31.7	70.8	28.2	0.0909
Aliphatic acids													
Tetradecanoic acid	58.9	37.2	40.9	44.5	40.8	44.4	52.4	43.6	48.7	42.5	41.5	43.3	0.7831
(Z)-9-Octadecenoic acid	40.1	25.9	32.0	29.7	23.7	29.5	42.3	31.6	22.6	29.4	24.0	33.7	0.2251
Sum	99.0	63.1	72.9	74.2	64.5	73.9	94.7	75.2	71.4	71.9	65.5	77.0	0.3121
Mono- and sesquiterpenes													

***(Z)*-Linalool oxide**	34.1	*abc*	25.3	*ab*	33.5	*abc*	20.8	*a*	66.0	*d*	39.5	*abc*	42.5	*bc*	38.1	*bc*	91.4	*e*	50.0	*cd*	60.9	*d*	49.5	*cd*	0.0001
(E)-Linalool oxide	35.2		28.8		34.0		28.7		41.7		29.5		33.9		33.0		34.5		28.8		31.6		30.3		0.8741
**Linalool oxide pyranoid**	33.0	*abcdef*	21.9	*ab*	32.5	*abcdef*	18.9	*a*	53.9	*gh*	31.9	*abcde*	43.0	*cdefg*	30.8	*bcd*	82.5	*i*	44.8	*dfgh*	55.2	*h*	46.9	*efgh*	0.0001
Linalool	nd		nd		nd		nd		10.2		6.5		5.7		4.8		23.3		10.6		21.4		11.1		0.1169
**Hotrienol**	28.4	*a*	23.2	*a*	26.9	*a*	10.1	*a*	96.6	*cd*	56.2	*abcd*	54.2	*abc*	37.4	*ab*	206	*e*	80.8	*bcd*	93.6	*d*	90.1	*cd*	<.0001
Nerol	16.1		5.4		9.6		7.9		10.0		8.5		15.3		6.0		34.4		16.3		36.3		13.7		0.1757
**Lavandulol**	15.7	*a*	15.8	*a*	14.0	*a*	16.5	*ab*	15.6	*a*	14.3	*a*	16.0	*a*	16.0	*a*	20.2	*ab*	16.8	*ab*	26.1	*b*	17.6	*ab*	0.0403
(E)-8-Hydroxylinalool	73.3		43.0		56.5		47.8		91.7		55.3		81.1		54.3		138.4		78.4		93.2		77.8		0.1451
***(Z)*-8-Hydroxylinalool**	482	*abcd*	211	*a*	391	*abc*	233.8	*ab*	667	*bcd*	399	*abc*	655	*bcd*	390	*abc*	1 369	*e*	630	*bcd*	851	*d*	692	*cd*	0.0215
Linalyl isobutyrate	28.2		20.3		22.1		22.5		30.4		24.8		30.7		27.3		38.3		29.3		25.2		28.1		0.8010
2,6-Dimethyl-2,6-Octadiene-1,8-diol	nd		nd		nd		nd		16.9		9.1		10.1		7.2		9.4		12.0		16.4		12.1		0.0763
Nerolidol	11.3		7.0		8.9		6.6		13.1		9.7		13.1		8.6		18.8		11.5		13.7		11.5		0.3623
**Lilac alcohol C**	4.8	*ab*	4.0	*ab*	4.7	*ab*	2.5	*a*	7.8	*bcd*	4.6	*ab*	8.7	*cde*	5.5	*abc*	19.4	*g*	11.2	*def*	14.9	*f*	12.5	*ef*	0.0001
Sum	762	*ab*	406	*a*	634	*ab*	416	*a*	1 121	*bc*	689	*ab*	1 009	*bc*	659	*ab*	2 086	*d*	1 021	*bc*	1 339	*c*	1 093	*bc*	0.0042
C_{13}-norisoprenoids																									
3-Hydroxy-*β*-damascone	160		136		142		107		172		147		166		144		184		161		149		174		0.3915
3-Hydroxy-7,8-dihydro-*β*-ionol	81.5		61.5		65.4		68.6		78.3		65.4		78.1		66.5		102		73.1		67.0		82.6		0.8125
3-Oxo-*α*-ionol	327		299		283		229		352		286		367		318		368		320		294		324		0.2457
β-ionol	252		168		165		175		206		147		207		161		252		152		148		157		0.5522
3-hydroxy-5,6-epoxy-*β*-ionone	23.2		19.6		20.4		19.9		21.8		17.1		26.7		17.9		28.5		24.3		24.3		24.8		0.2484
3-Oxo-7,8-dihydro-*α*-ionol	248		204		200		200		227		181		236		204		233		191		183		199		0.2617

Dihydro-3-oxo-*β*-ionol	22.0		12.3		17.1		17.4		19.2		16.2		19.6		16.5		23.2		17.6		18.5		21.3		0.7504
Sum	1 113		900		892		817		1 077		860		1 100		928		1 190		938		884		983		0.4356
Volatile phenols																									
p-Vinylguaiacol	23.1		17.2		22.3		15.2		18.1		14.2		20.7		14.8		22.6		19.9		16.7		19.1		0.8246
Eugenol	35.1		32.5		30.6		32.8		39.0		27.4		40.6		46.7		35.9		32.8		39.1		54.0		0.6812
Methoxyeugenol	11.2		11.8		9.3		9.4		10.3		9.3		10.0		9.9		10.9		11.7		12.9		10.2		0.5202
2-Hydroxy-benzeneethanol	13.5		8.2		11.0		8.2		11.5		6.9		10.0		9.9		31.3		19.4		21.3		11.5		0.2871
Isoeugenol	24.9		10.7		18.9		11.5		17.3		8.7		17.3		10.4		25.0		11.6		13.7		10.5		0.7023
Isovanillyl alcohol	36.2		17.4		30.6		18.2		26.6		15.7		32.7		17.1		37.9		23.2		21.9		24.3		0.4105
Acetovanillone	33.8		25.9		26.6		23.3		26.0		21.8		32.3		21.2		32.5		28.2		28.9		28.5		0.0533
**Methyl vanillate**	76.5	*a*	42.6	*a*	62.9	*a*	55.9	*a*	68.4	*a*	46.4	*a*	83.4	*ab*	42.7	*a*	242	*b*	113	*ab*	59.2	*a*	62.7	*a*	0.0423
Methyl 3-hydroxybenzoate	29.0		19.2		33.9		20.2		32.6		20.5		26.5		21.3		34.5		27.4		24.2		20.2		0.2786
(E)-Coniferyl alcohol	65.9		32.6		36.9		22.7		40.5		23.4		55.4		19.7		62.8		33.1		35.5		30.3		0.5081
Sinapyl alcohol	39.4		18.5		21.8		17.4		25.2		18.3		26.3		15.7		38.3		21.1		19.8		17.9		0.8714
Salicyl alcohol	17.2		15.1		15.8		12.6		18.7		19.0		13.6		16.7		27.0		22.3		19.2		15.0		0.5949
5-(3-Hydroxypropyl)-2,3-dimethoxyphenol	11.5		9.3		8.8		7.3		13.0		7.4		14.2		6.3		17.2		11.7		9.7		9.0		0.6950
2-Hydroxy-4,5-dimethylacetophenone	36.2		34.1		29.6		32.3		44.6		28.7		35.2		30.9		39.3		33.7		29.8		31.7		0.7859
4-tert-Butyl-2-methylphenol	54.0		47.4		42.3		50.9		55.5		43.3		50.7		46.5		51.1		49.2		43.2		49.3		0.8176
Sum	508		342		401		338		447		311		469		330		708		458		395		394		0.3361
Benzene derivatives																									
Benzyl alcohol	1 098		1 410		983.6		1 229		1 101		1 257		1 027		1 345		1 082		1 503		1 002		1 351		0.5063

2-Phenylethanol	752	1 143	742	1 048	790	1 090	745	1 141	767	1 225	781	1 179	0.8129
3-Tridecyl ester-*m*-toluic acid	87.7	78.3	71.2	122.6	84.3	70.2	96.1	71.3	86.2	67.3	69.6	81.6	0.4832
4-Benzyloxy-3-methoxybenzyl alcohol	24.3	25.3	17.0	22.5	21.3	26.3	17.6	23.2	22.8	33.7	16.4	19.9	0.5483
Sum	1 961	2 656	1 814	2 422	1 997	2 443	1 885	2 580	1 958	2 828	1 869	2 631	0.6319
Other volatiles													
2-Butyltetrahydro-furan	8.2	6.7	6.9	6.4	9.0	7.5	8.2	8.0	9.9	9.7	9.9	10.0	0.7505
**5-(2-Tetrahydrofurfuryl)-heptan-2-ol**	24.7 *abcde*	14.6 *ab*	20.1 *abcd*	11.2 *a*	34.0 *abcde*	20.8 *abcde*	35.8 *bcde*	19.2 *abc*	85.3 *f*	37.6 *cde*	44.2 *e*	39.6 *de*	0.0001
6-Ethenyl-2,2,6-trimethyloxan-3-ol	35.0	28.8	35.3	31.0	38.7	28.9	34.4	31.6	35.3	29.4	35.0	31.2	0.9595
Total	4 701	4 596	4 036	4 251	4 971	4 589	4 786	4 809	6 390	5 601	4 918	5 438	0.5483

[a]All compounds were quantified as 2-octanol equivalents. Data are means of n=5.For each compound, values with different letters indicate significant differences according to Tukey's test at p<0.05, in terms of interaction. ns: not significant; nd: not determined.

2 Supplementary Figures

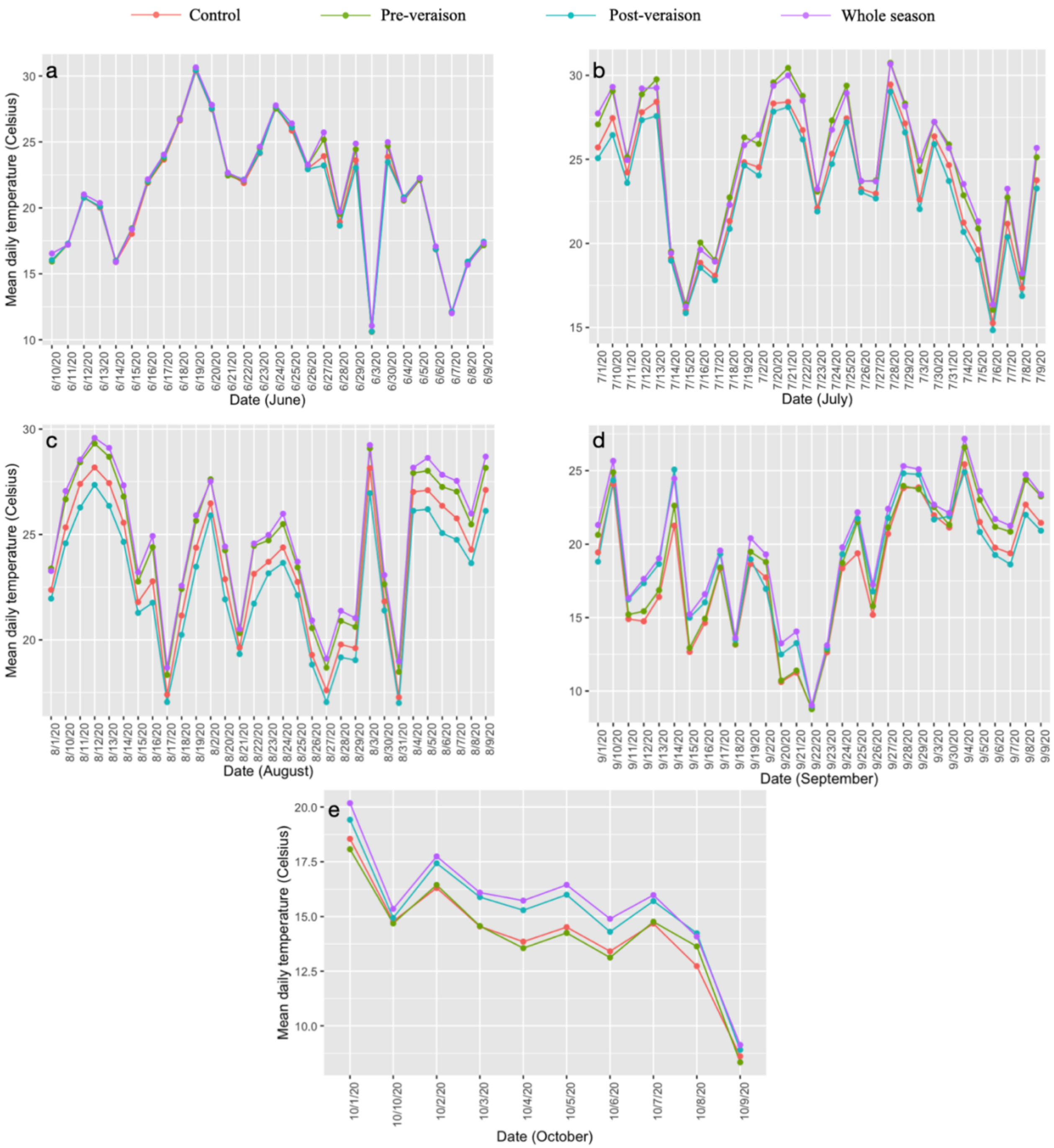

Supplementary Figure 1. Mean temperature changes in the treatments (CT (control), PRE (pre-veraison), PT (post-veraison) and W (whole season)) during 2020 during the 5 months of trial (June(a), July (b), August (c), September (d), October (e)). Values showed are means of 4 to 5 data loggers per treatment (Temperature: n=5 for W, PRE and PT; n=4 for CT).

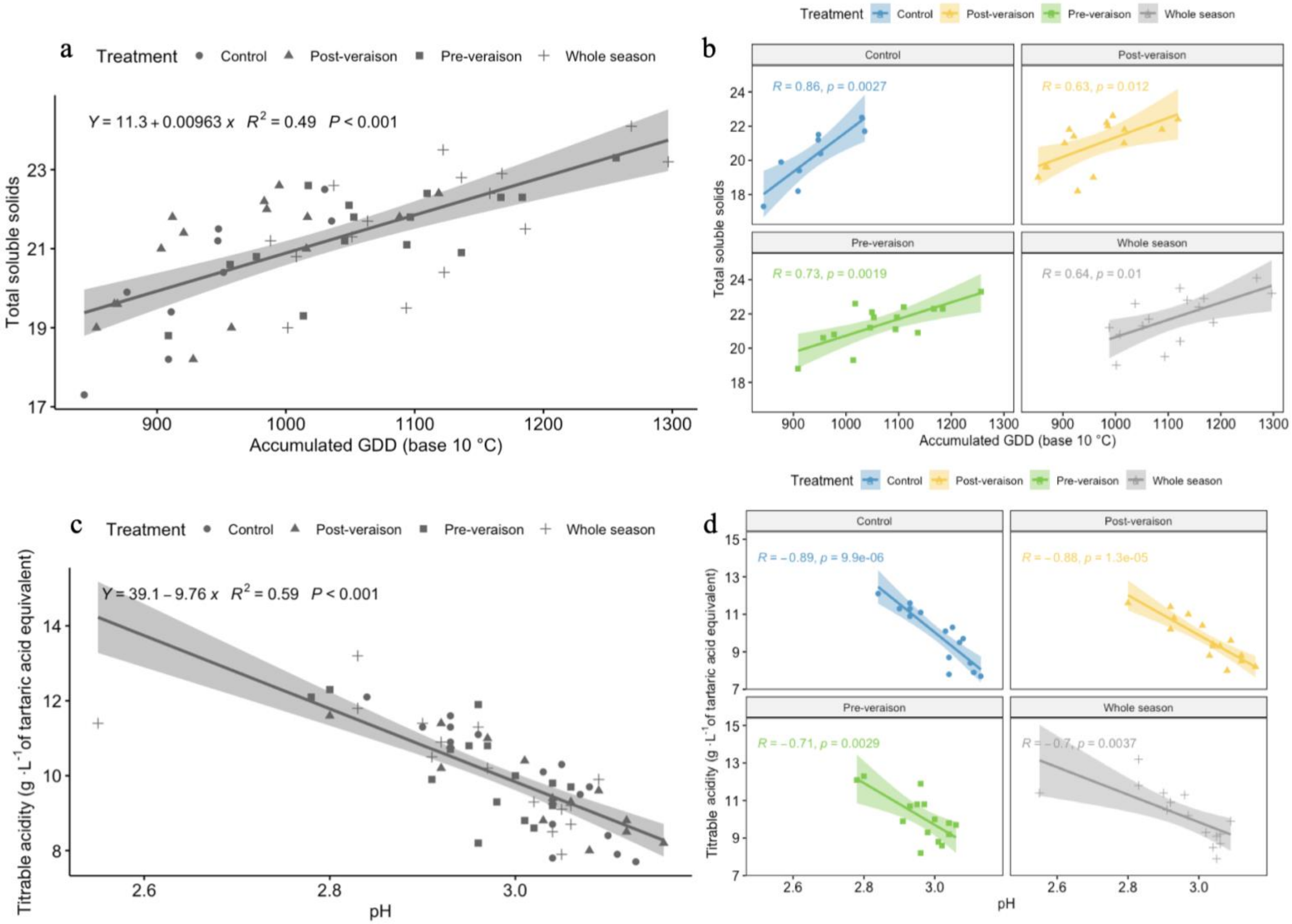

a
Treatment Control Post-veraison Pre-veraison Whole season
Y = 11.3 + 0.00963 x R² = 0.49 P < 0.001
Total soluble solids
Accumulated GDD (base 10 °C)
b
Treatment Control Post-veraison Pre-veraison Whole season
Control
Post-veraison
Pre-veraison
Whole season
R = 0.86, p = 0.0027
R = 0.63, p = 0.012
R = 0.73, p = 0.0019
R = 0.64, p = 0.01
Total soluble solids
Accumulated GDD (base 10 °C)
c
Treatment Control Post-veraison Pre-veraison Whole season
Y = 39.1 − 9.76 x R² = 0.59 P < 0.001
Titrable acidity (g·L⁻¹of tartaric acid equivalent)
pH
d
Treatment Control Post-veraison Pre-veraison Whole season
Control
Post-veraison
Pre-veraison
Whole season
R = −0.89, p = 9.9e-06
R = −0.88, p = 1.3e-05
R = −0.71, p = 0.0029
R = −0.7, p = 0.0037
Titrable acidity (g·L⁻¹of tartaric acid equivalent)
pH

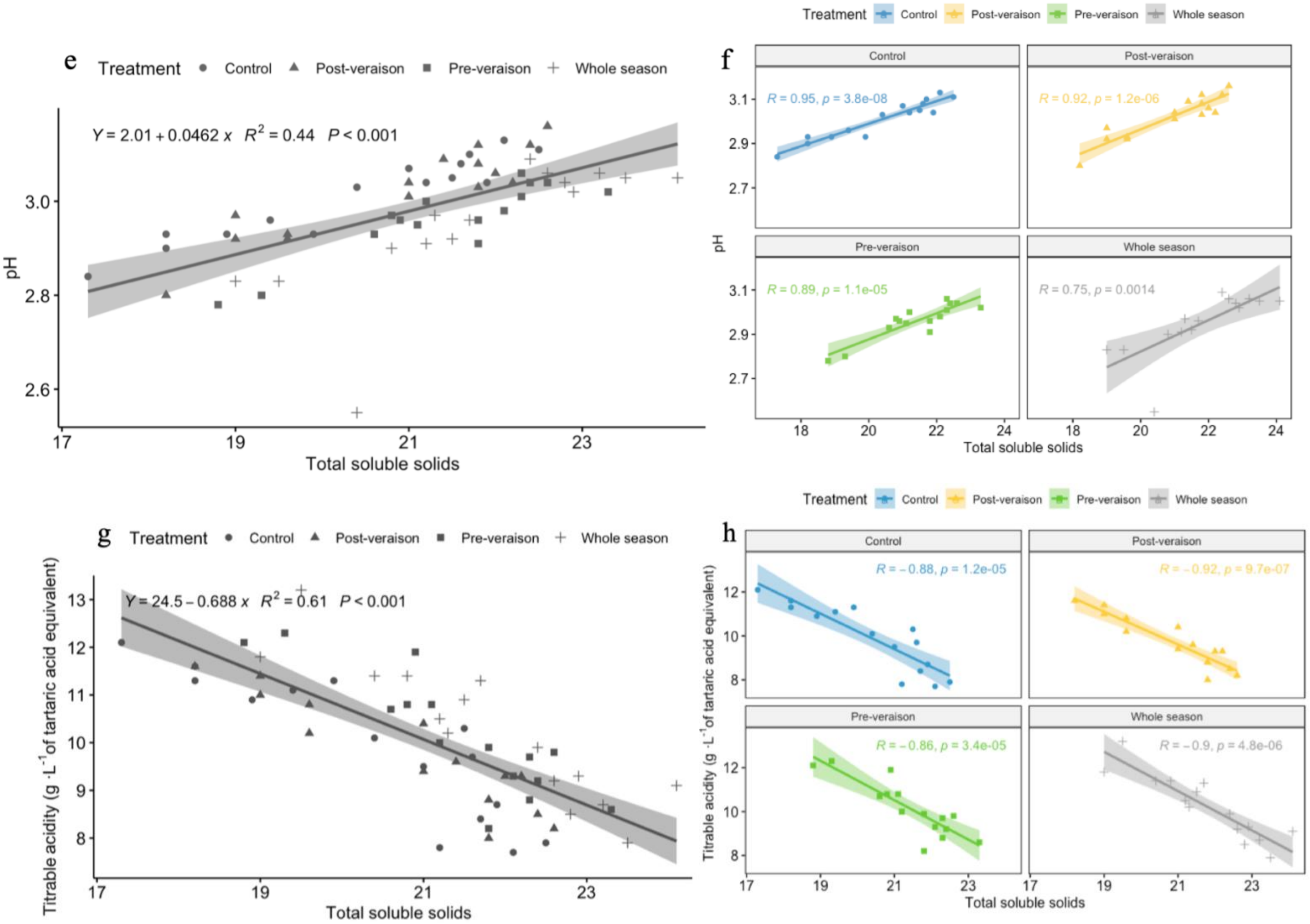

Supplementary figure 2. Linear relationships between different parameters. Accumulated GDD and Total Soluble Solids: (a) shows the linear relation using all data (b) shows the values for each treatment. pH and Titratable acidity (g · L^{-1} tartaric acid eq): (c) shows the linear relation using all data (d) shows the values for each treatment. Total Soluble Solids and pH: (e) shows the linear relation using all data (f) shows the values for each treatment. Titratable acidity (g · L^{-1} tartaric acid eq) and Total Soluble Solids: (g) shows the linear relation using all data (h) shows the values for each treatment.

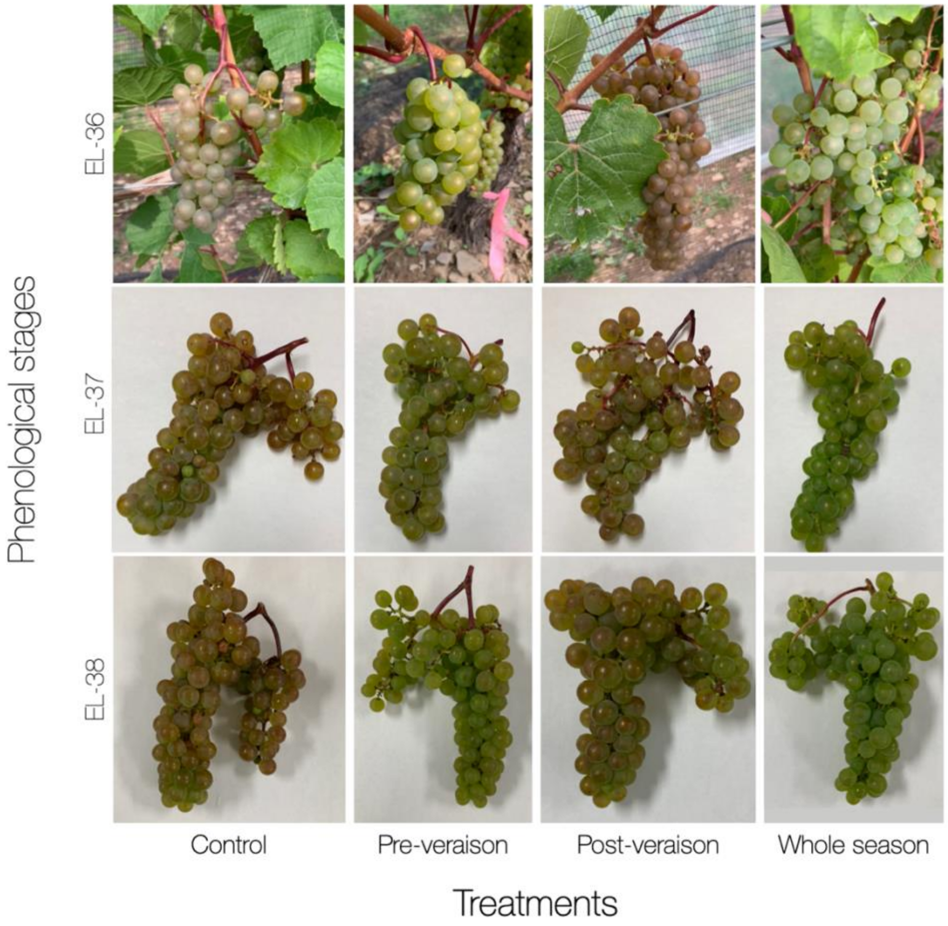

Supplementary figure 3. Visual aspect of grape clusters from the mini-greenhouse treatments (Control, Pre, Post, Whole) at three phenological stages (EL-36, EL-37, EL-38). (Pictures: F. Campos).
